# Enhancing WRAP‐Based Nanoparticles for Small Interfering Ribonucleic Acid Delivery in pH‐Sensitive Environments

**DOI:** 10.1002/cmdc.202400885

**Published:** 2025-04-10

**Authors:** Giulia Di Gregorio, Coélio Vallée, Karidia Konate, Clémentine A. Teko‐Agbo, Thania Hammoum, Héloïse Faure‐Gautron, Yannick Bessin, Sebastien Deshayes, Eric Vivès, Albano C. Meli, Pascal de Santa Barbara, Sandrine Faure, Stéphanie Barrère‐Lemaire, Sébastien Ulrich, Prisca Boisguérin

**Affiliations:** ^1^ PhyMedExp University of Montpellier, INSERM, CNRS 371 Av. Doyen Giraud 34295 Montpellier France; ^2^ IBMM Institut des Biomolécules Max Mousseron University of Montpellier, CNRS, ENSCM 1919 route de Mende 34293 Montpellier France; ^3^ Institut de Génomique Fonctionnelle University of Montpellier, CNRS, INSERM CNRS 141 rue de la Cardonille 34293 Montpellier France

**Keywords:** acylhydrazones, peptide‐based nanoparticles, pH sensitive, small interfering ribonucleic acid delivery, targeting

## Abstract

Small interfering RNAs (siRNA) are promising therapeutic molecules that require delivery systems to reach their targets. Several siRNA delivery systems, such as lipid‐ or peptide‐based nanoparticles, are developed for different pathologies. In this context, we previously conceived a cell‐penetrating peptide WRAP5‐forming nanoparticles in the presence of siRNAsand validated the efficiency of this delivery system in inhibiting protein expression. In the pathophysiological context of acute myocardial infarction, which causes a pH drop in the ischemic heart tissue, we optimized the WRAP5‐based nanoparticles for a pH‐sensitive siRNA‐targeted delivery. Therefore, pH‐sensitive acyl hydrazone linkers are used to graft polyethylene (PEG) on the WRAP5 peptide. Proof of concept of the targeted delivery is performed using siRNA silencing the Fas‐associated death domain (FADD)‐containing protein implicated in apoptosis during myocardial ischemia‐reperfusion injury on two human cell models (vascular endothelial cells and hiPSC‐derived cardiomyocytes). The results show that only PEGylated WRAP5 nanoparticles via an appropriate acyl hydrazone linker can induce a specific FADD knockdown at pH 5 compared to naked nanoparticles. These optimized WRAP‐based nanoparticles could be a novel therapeutic tool for treating myocardial infarction by inhibiting apoptosis induced by reperfusion and maximizing local delivery of the nanoparticle content at the site of injured cells.

## Graphical abstract

PEGylation of the WRAP5‐based nanoparticles via acyl hydrazone linkers is achieved to obtain a pH‐sensitive small interfering ribonucleic acid‐targeted delivery. In acidic extracellular conditions (pH 5) occurring during myocardial ischemia‐reperfusion injury, this optimized delivery system is the only one showing efficient silence of the Fas‐associated death domain‐containing protein implicated in apoptosis, as exemplified in two human cell models.

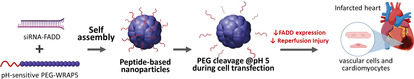

## 1 Introduction

More and more pharmaceutical agents using nucleic acids have emerged in the market, exemplified by the development of six approved small interfering ribonucleic acid (siRNA)‐based drugs by the U.S. Food and Drug Administration since 2018.^[^ ^1^ ^]^ However, despite the enormous therapeutic potential, the main challenge remains an efficient delivery of these therapeutic molecules to the site of action, as cell membranes act as biological structures ensuring cell integrity by separating the internal contents from the external medium. All drugs with an intracellular target must cross this phospholipid bilayer matrix.^[^ ^2^, ^3^ ^]^ A growing community of researchers has focused on membrane‐active compounds that perturbate cell membranes to activate the transport of different drugs.^[^ ^4^ ^]^ Among these, cell‐penetrating peptides are promising tools, especially amphipathic peptides with structural versatility that often translate into favorable physicochemical and biochemical properties. Several proofs of concept have been published showing in vitro and in vivo applicability of the peptide‐based delivery systems able to form nanoparticles in the presence of siRNA as reported for peptide families such as C6M1, PepFect, RALA, etc.^[^ ^5^, ^6^, ^7^ ^]^

In this context, we have conceived W‐ and R‐rich amphipathic peptides (WRAP) composed of only three amino acids (leucine, arginine, and tryptophan).^[^ ^8^ ^]^ WRAP‐based nanoparticles loaded with siRNA internalize cells within 15 min of incubation mainly, through direct translocation, bypassing sequestration in endosomes.^[^ ^9^ ^]^ Up to 90% knock‐down efficiency of different types of endogenous proteins is achieved at siRNA concentrations between 20 and 50 nM.

In the present study, we aimed to develop a potent cardioprotective therapy to treat reperfusion injury observed during artery reopening in acute myocardial infarction (AMI). Indeed, AMI is among cardiovascular diseases the number one killer worldwide. In Europe, 500,000 AMI patients die every year.^[^ ^10^ ^]^ The standard of care to save AMI patients is to reopen, as soon as possible, the occluded artery mainly using primary coronary angioplasty. Prompt revascularization of the ischemic myocardium has dramatically improved functional recovery and patient survival.^[^ ^11^, ^12^ ^]^ However, the abrupt return of oxygenated blood in the weakened ischemic region causes additional lesions (programmed cell damage) called reperfusion injury. Although most patients are doing well after the intervention, in the long term, some patients undergo left ventricle remodeling leading to life‐threatening chronic cardiac pathologies. Unfortunately, in 2024, there is still no treatment that specifically abolishes myocardial reperfusion injury.

Cardiomyocytes and endothelial cells are the two main and abundant cell types of the heart, with 30% of the cardiac and 50% endothelial cells.^[^ ^13^ ^]^ During pathological situations such as ischemia‐reperfusion injury, these two cell types undergo death through inflammation, oxidative stress, and calcium overload^[^ ^14^ ^]^ with microvascular endothelial cell injury occurring much earlier and with much greater severity than cardiomyocyte injury.^[^ ^15^ ^]^ Hemorrhages in the cardiac muscle are associated with severe ischemic injury during coronary occlusion.^[^ ^16^, ^17^ ^]^

During ischemia‐reperfusion injury, the crucial role of the extrinsic apoptotic pathway mediated by the first apoptosis signal (FAS) receptor (tumor necrosis factor receptor superfamily member 6, also known as APO‐1/CD95) has been demonstrated in the murine heart.^[^ ^18^ ^]^ Triggered by the ischemic stress, the adaptor protein Fas‐associated protein with death domain (FADD) interacts with the intracellular region of the FAS receptor, coupling the extracellular signal to the intracellular apoptotic machinery.^[^ ^19^ ^]^ The important role of FADD in myocardial reperfusion injury was further underlined by the fact that genetic FADD inactivation (dominant negative strategy) significantly reduced infarct size and improved cardiac function and survival in mice.^[^ ^20^ ^]^ Therefore, our main objective was to propose an innovative strategy for AMI treatment that will specifically target the FADD‐dependent apoptotic pathway using siRNA‐FADD encapsulated in our WRAP‐based nanoparticles validated as siRNA delivery system.^[^ ^8^, ^21^, ^22^, ^23^ ^]^

Because it is well known that during ischemic stress, an extracellular pH drop to a value of 5 is observed in the myocardium,^[^ ^24^, ^25^ ^]^ we aimed in this report to use a dynamic PEGylation approach that involves the insertion of a pH‐sensitive linker between the PEG moiety and the WRAP peptide. PEGylation of the nanoparticle is essential for ensuring its stability and facilitating intravenous injection for in vivo administration (reduction of interaction with serum proteins). However, it should be noted that PEGylation can mask the nanoparticle, thereby reducing its cellular internalization. Therefore, our strategy was to obtain optimized nanoparticles for the siRNA delivery to ischemic tissues, where the PEG moiety will be cleaved due to the ischemic pH, thus enabling targeted cellular internalization.

In detail, the present work reported the PEGylation of the WRAP5 peptide using pH‐sensitive hydrazone linkages. The subset of synthesized WRAP5 conjugates was evaluated for their ability to form nanoparticles in the presence of siRNA targeting the FADD protein. Internalization and FADD silencing in human vascular endothelial cells (EA.hy926) and human‐induced pluripotent stem cell (hiPSC)‐derived cardiomyocytes were evaluated at pH 7.4 and pH 5. Our results demonstrate that nanoparticles formed with the PEGylated WRAP5 peptide bearing a finely tuned acyl hydrazone pH‐sensitive linker are suitable for siRNA delivery under these acidic conditions compared to the naked WRAP5 nanoparticles. PEG‐HyOMe‐W5 could be a suitable delivery vector for nucleic acid delivery in ischemic tissues after AMI using intravenous or intracardiac administration routes.

## 2 Results and Discussion

### 2.1 Synthesis and Characterization of pH‐Sensible PEGylated WRAP5 Conjugates

Acyl hydrazones are well‐known pH‐sensitive groups that are stable at physiological pH but undergo hydrolysis at mild acidic pH. Our group has thus used these chemical entities to design degradable nucleic acid delivery vectors.^[^ ^26^, ^27^, ^28^, ^29^, ^30^ ^]^ However, one limitation of acyl hydrazones is their slow hydrolysis rate, and methodologies that enable tuning their hydrolysis kinetics are still lacking. In this work, we explored the effect of neighboring group participation in the rate of hydrolysis as the grafted entity on the WRAP‐based nanoparticle should be rapidly cleaved to allow cellular internalization.

**Figure 1:**
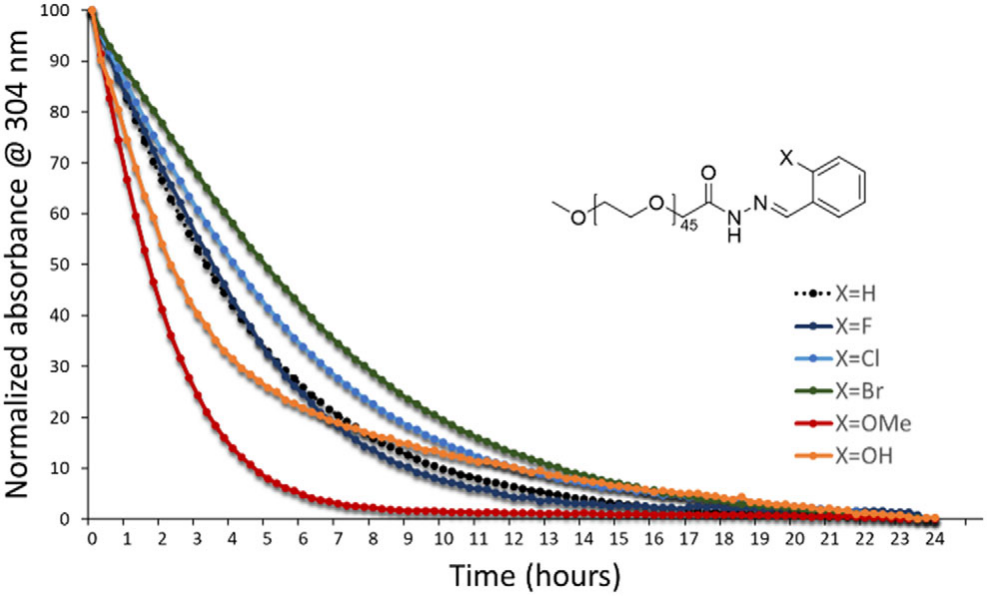
Hydrolysis kinetics of model PEG acyl hydrazone conjugates. Molecular structures (left) and kinetic of hydrolysis at pH 4 (normalized UV–vis absorption monitored at 304 nm, the samples were prepared at 50 μM of acyl hydrazone conjugate, right).

Such a rate enhancement has been documented for forming acyl hydrazone‐based conjugates at neutral pH with boronic acids^[^ ^31^, ^32^, ^33^, ^34^, ^35^ ^]^ and halogen^[^ ^36^ ^]^ neighboring groups placed near the reactive aldehyde partner. N— or O—hydrogen bond acceptors^[^ ^37^, ^38^, ^39^ ^]^ have also been shown to accelerate the formation of the related hydrazone‐ and oxime‐bond conjugates.^[^ ^38^, ^39^, ^40^, ^41^ ^]^ However, little is known about the effect of such neighboring groups on the pH sensitivity of acyl hydrazone conjugates. We have thus prepared conjugate models of mPEG2000 acyl hydrazone featuring varying neighboring groups (H, F, Cl, Br, OH, OMe) selected from commercially available benzaldehyde derivatives (**Figure** **1**, for more details on the synthesis, refer to Figure S1 and S2, Supporting Information).

Their hydrolysis kinetics at various pH in aqueous buffers were then studied by UV–vis spectroscopy, monitoring the decrease of the specific acyl hydrazone absorbance band at 304 nm. While no significant hydrolysis was detected at neutral pH over 48 h (data not shown), the hydrolysis kinetics were found to vary significantly at acidic pH for the different neighboring groups. The half life of the model conjugates increases gradually down the halogen series from 3–4 h (X = H) to 5 h (X = Br) and decreases to 2–3 h (X = OH, OMe) at pH 4 (Figure 1). The trend was similar at pH 5, with the shortest half life of 5–6 h for X = OMe (Figure S3, Supporting Information). From these results, most visible at pH 4 (Figure 1), we selected the methoxy group as the fastest hydrolysis enhancer.

**Scheme 1:**
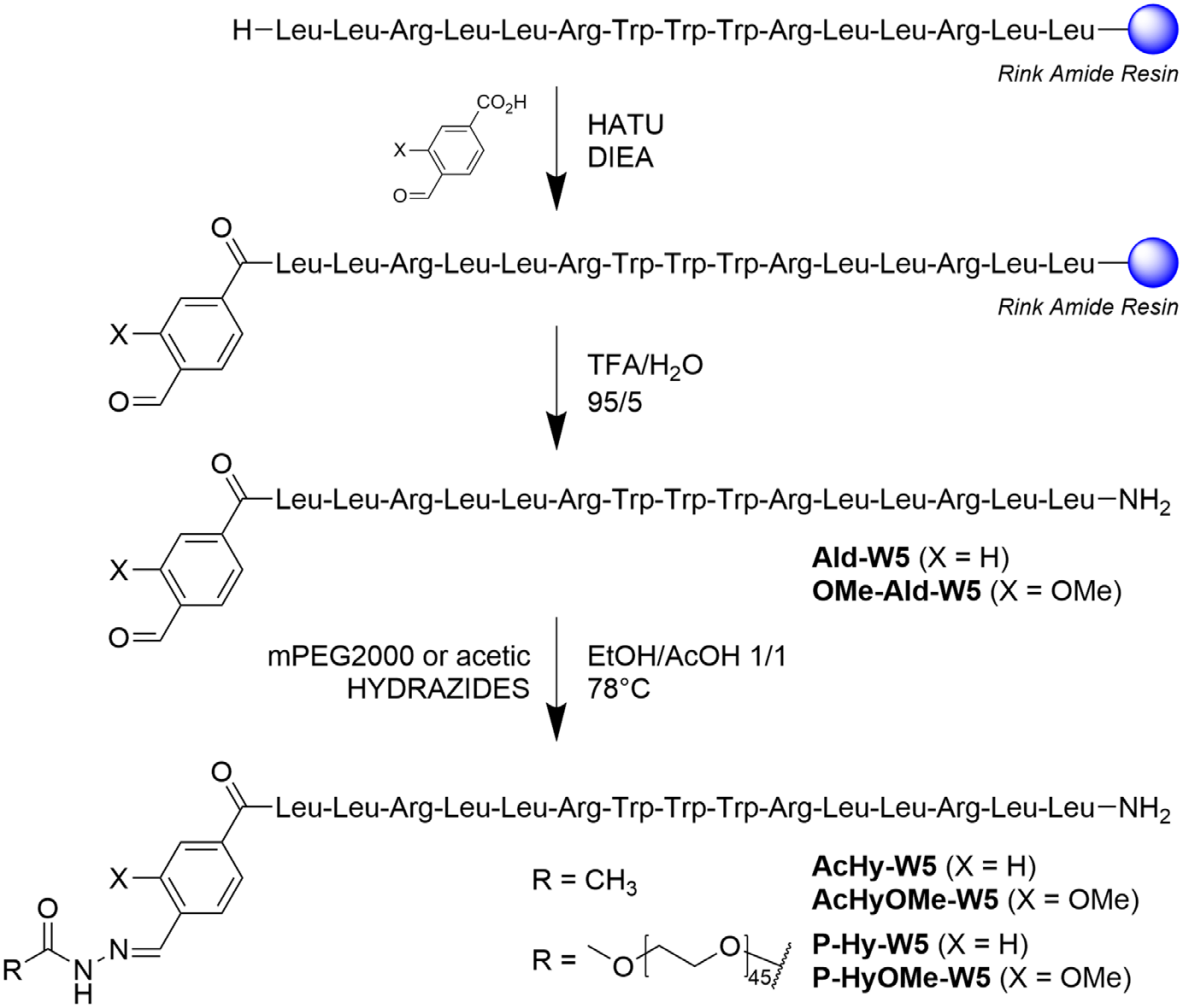
Synthetic route showing the preparation of the functionalized WRAP5 peptides and their acyl hydrazone conjugates.

We then proceeded with the synthesis of the corresponding PEG‐WRAP5 conjugates P‐Hy‐W5 and P‐HyOMe‐W5 which were prepared by coupling mPEG2000 with Ald‐W5 and AldOMe‐W5 (**Scheme** **1**, for more details on the synthesis, refer to Figure S4 and S5, Supporting Information). AcHyd‐W5 was also prepared as a control compound.

**Figure 2:**
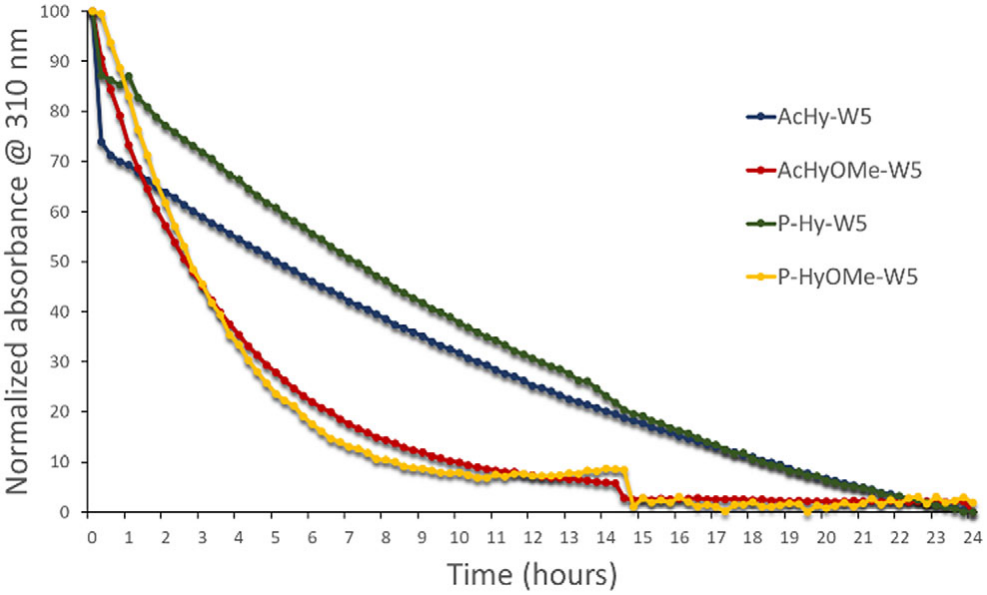
Kinetic of hydrolysis at pH 4 (normalized UV–vis absorption monitored at 310 nm). The samples were prepared at 25 μM of acyl hydrazone conjugates.

The pH sensitivity of these conjugates (AcHy‐W5, AcHy‐OMe‐W5, P‐Hy‐W5, and P‐Hy‐OMe‐W5) was then similarly investigated by UV–vis spectroscopy at 37 °C, monitoring the evolution of the acyl hydrazone absorbance band at 310 nm at different pH. Again, no hydrolysis was detected at neutral pH over 48 h (Data not shown). In acidic buffers, the presence of the PEG seems to hinder slightly the hydrolysis process, most likely due to shielding of the acyl hydrazone motif from the bulk solvent, thus resulting, at pH 4, in an increase of half lives from 5–6 h for AcHy‐W5 to 7–8 h for P‐Hy‐W5. However, the main conclusion from the data obtained is that the neighboring OMe group accelerates the hydrolysis of the WRAP conjugates, thus confirming the preliminary data obtained with the model acyl hydrazone conjugates. Half lives at pH 4 of 2–3 h for AcHyOMe‐W5 versus 5–6 h for AcHy‐W5 and of 2–3 h for P‐HyOMe‐W5 versus 7–8 h for P‐Hy‐W5 were thus determined (**Figure** **2**).

### 2.2 Optimization of the Formulation Conditions for PEGylated WRAP5 Nanoparticles

Four siRNAs designed to interact at different regions along the FADD mRNA (Table S1, Supporting Information) were selected based on alignments confirming low off‐targets (data not shown). The silencing properties of the four siFADD were first evaluated at the same concentration using the naked WRAP5‐based nanoparticle in human vascular endothelial cells (EA.hy296 cells) (Figure S6A, Supporting Information). The siFADD‐3 (siF3) showed the highest FADD silencing (>60% at 40 nM), which was further confirmed using a dose‐dependent incubation compared to siFADD‐2 (≈30% silencing at 40 nM), as exemplified by Western blot evaluation (Figure S6B,C, Supporting Information). siFADD‐3 was selected for all experiments performed in vitro (dynamic light scattering (DLS) measurements or on cells), as it has the highest FADD silencing efficiency.

Next, we evaluated the ability of PEG‐Hy‐WRAP5 (P‐Hy‐W5) and PEG‐HyOMe‐WRAP5 (P‐HyOMe‐W5) to form nanoparticles in the presence of siFADD‐3 in comparison to WRAP5 or intermediate products such as Ald‐W5 or AcHy‐W5. Since the PEG functionalization increases the hydrophobicity of the WRAP peptide due to the loss of the N‐terminal amine group, resulting in lower solubility, we solubilized the grafted WRAP5 peptides alone in water, 20% ethanol/H_2_O, or 20% acetonitrile/H_2_O. The nanoparticles were formulated in an aqueous solution containing 5% glucose, and the solvent's effect on the nanoparticle formation was evaluated by DLS measurements (see **Table** **1** and S2, Supporting Information).

**Table 1:** DLS characterization of the WRAP5‐based nanoparticles used in the study.

Nanoparticlesa)	Size [nm]	PdIb)	ZP [mV]c)
W5:siF3	85.7 ± 9.8	0.268 ± 0.034	36.9 ± 4.9
PW5:siF3d)	91.6 ± 6.5	0.213 ± 0.039	29.6 ± 2.9
Ald‐W5:siF3	86.8 ± 6.0	0.314 ± 0.045	34.4 ± 2.4
AcHy‐W5:siF3	115.5 ± 35.3	0.293 ± 0.055	n.d.e)
P‐Hy‐W5:siF3	94.2 ± 10.8	0.273 + 0.043	−6.9 ± 1.9
P‐HyOMe‐W5:siF3	103.6 ± 16.2	0.301 ± 0.064	24.3 ± 3.1

a) Peptides were solubilized in 20% ethanol. Nanoparticles were formulated in an aqueous solution containing 5% glucose (Peptide:siRNA molar ratio 20, siRNA concentration of 500 nM);

b) PdI = Polydispersity Index;

c) Zeta potential (ZP) values were acquired in the presence of NaCl (2 mM);

d) PW5 = PEGylated WRAP without pH‐sensitive linkage;

e) n.d. = not determined.

Nanoparticles formulated in water using the WRAP5 conjugates revealed the biggest sizes (around 100–140 nm) with more heterogenicity (polydispersity indexes around 0.4) as expected, probably due to their lower solubility. Compared to that, WRAP5:siF3 nanoparticles are in the range of previously published values (mean size: 92.7 ± 19.4 nm, PdI: 0.275 ± 0.071, Table S2, Supporting Information).^[^ ^8^ ^]^

The same tendency for bigger nanoparticles is observed for all conditions using the peptides solubilized in 20% acetonitrile/H_2_O before the nanoparticle formulation (mean size > 140 nm, PdI around 0.4, Table S2, Supporting Information).

In contrast, if the WRAP5 conjugates were solubilized in 20% EtOH/H_2_O before the nanoparticle formulation at room temperature, we measured mean sizes around 100 nm with PdIs around 0.3 or lower for all nanoparticles (Table 1). In detail, compared to the naked WRAP:siF3 nanoparticles, we obtained similar nanoparticles with PEG‐WRAP5 (PW5 without pH linkage), PEG‐Hy‐W5, PEG‐HyOMe‐W5, Ald‐W5, and AcHy‐W5, showing that neither the PEG moiety nor the pH‐sensitive linkage has a negative impact on the formation of the nanoparticles.

Moreover, the chosen formulation conditions seemed optimal since W5:siF3 and PEG‐HyOMe‐W5:siF3 nanoparticles were stable over 7‐day storage at 4 °C (Table S3, Supporting Information).

Finally, we evaluated the effect of the temperature on the nanoparticle formulation by comparing only W5:siF3 and PEG‐HyOMe‐W5:siF3. For that, we performed the peptide and siRNA mixture at 4 or 37 °C (Table S3, Supporting Information). At low temperatures (blue lines), we obtained heterogeneous nanoparticles with big mean sizes or two distinguished populations. In contrast, a higher temperature (red lines) had less impact on the formulation, as the nanoparticles had nearly the same size and PdI as those prepared at room temperature.

### 2.3 The Activity of pH‐Sensitive WRAP5 Nanoparticles in Endothelial Cells Depends on the PEGylation Percentage

**Figure 3:**
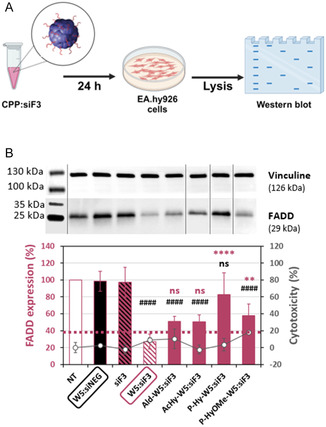
Evaluation of the FADD silencing property depending on the used grafted W5:siRNA nanoparticles. A) Scheme of the nanoparticle transfection on human endothelial EA.hy926 cells (by BioRender). B) Graphical representation of FADD expression in EA.hy926 cells treated with nanoparticles detected by Western blot. The dashed black lines indicate where images from different gels were joined. Graphical data represent mean ± SD, with *n* = 3 independent experiments in duplicates. Conditions: Nanoparticles with *R* = 20, siRNA = 40 nM/the dotted red line indicates 40% of FADD expression and the tolerated threshold of 20% cytotoxicity/negative controls: W5:siNEG (siRNA without a target) or siF3 alone. Statistical analysis: One‐way ANOVA with Turkey post‐test, ns > 0.05, ** = 0.001, and **** < 0.0001 versus W5:siF3 (pink square), or ns > 0.05, #### < 0.0001 versus W5:siNEG (black square).

According to the results presented in Table 1, we focused our investigation on a comparative study of silencing properties of the different pH‐sensitive WRAP conjugates with the naked WRAP5:siF3 nanoparticles. For this purpose, all analyzed nanoparticles were formulated in the same way (molar ratio (*R*) = 20, siRNA = 40 nM) and incubated on human endothelial EA.hy926 cells first in serum‐free medium for 90 min before supplementing the medium with 10% serum (**Figure** **3**A). This transfection protocol was selected because it has been previously validated for the optimal screening of siRNA‐loaded WRAP‐based nanoparticles.^[^ ^8^, ^9^, ^42^ ^]^ After 24 h transfection, the cells were lyzed and the FADD level was analyzed by Western blot (Figure 3B). As expected, WRAP5 encapsulating siRNA without cell target (siNEG, used as a negative control) or the siF3 alone showed no reduction in FADD expression. In contrast, the naked WRAP5:siF3 nanoparticles induced an important FADD silencing of around 70% as shown previously in Figure S6B, Supporting Information, highlighting the specificity of the delivery system. Concerning the grafted nanoparticles, we demonstrated that Ald‐W5:siF3 and AcHy‐W5:siF3 nanoparticles have a knockdown efficiency of 50%, not significantly different from W5:siF3, confirming that the pH‐sensitive linker did not influence the nanoparticle activity.

For the PEGylated WRAP5 nanoparticles, we found that PEG‐Hy‐WRAP5 had nearly no silencing effect (<20%, ns vs W5:siNEG). This was in accordance with the fact that the full PEGylation of peptide‐based nanoparticles masks the positive charge and hinders cellular internalization as previously demonstrated for PepFect and RICK peptides^[^ ^23^, ^43^ ^]^ but also polyplex delivery systems.^[^ ^44^ ^]^ Reduction of the surface charges due to the PEGylation was reflected by the zeta potential value decreased for P‐Hy‐W5:siF3 (ZP = −6.9 ± 1.9 mV) and P‐HyOMe‐W5:siF3 nanoparticles (ZP = 24.3 ± 3.1 mV) compared to the naked WRAP5:siF3 (ZP = 36.9 ± 4.9 mV) (Table 1). However, the significant difference observed between the ZP of the two PEGylated nanoparticles was unexpected. While the negative surface charge of the P‐Hy‐W5:siF3 nanoparticles might result from structural rearrangements, ion adsorption, or surface chemistry effects, the precise mechanism remains elusive and warrants further investigation.

Curiously, the P‐HyOMe‐W5:siF3 nanoparticles induced a FADD knockdown of ≈40% (^#### ^< 0.0001 vs W5:siNEG), which is nevertheless weaker than with W5:siRNA (** < 0.001). To explain this phenomenon, we compared the stability of P‐HyOMe‐W5:siF3, P‐W5:siF3, and W5:siF3 nanoparticles in the presence of heparin using a gel shift assay. Indeed, heparin (a negatively charged polymer) is a common compound used in peptide‐based nanoparticle research to analyze nanoparticle stability as it competes with the negatively charged siRNA for peptide binding.

As highlighted in Figure S7, Supporting Information, P‐W5:siF3 nanoparticles tolerated higher heparin concentrations compared to the naked W5:siF3 (100 μM vs 25 μM, respectively). On the other hand, the P‐HyOMe‐W5:siF3 nanoparticles were the most sensitive to heparin, destabilization occurring at a low heparin concentration of 12.5 μM. The difference obtained for P‐W5 and P‐HyOMe‐W5 suggests that the pH‐sensitive linker could impact the nanoparticle stability, enabling faster siF3 release and thus a better FADD silencing in endothelial cells.

**Figure 4:**
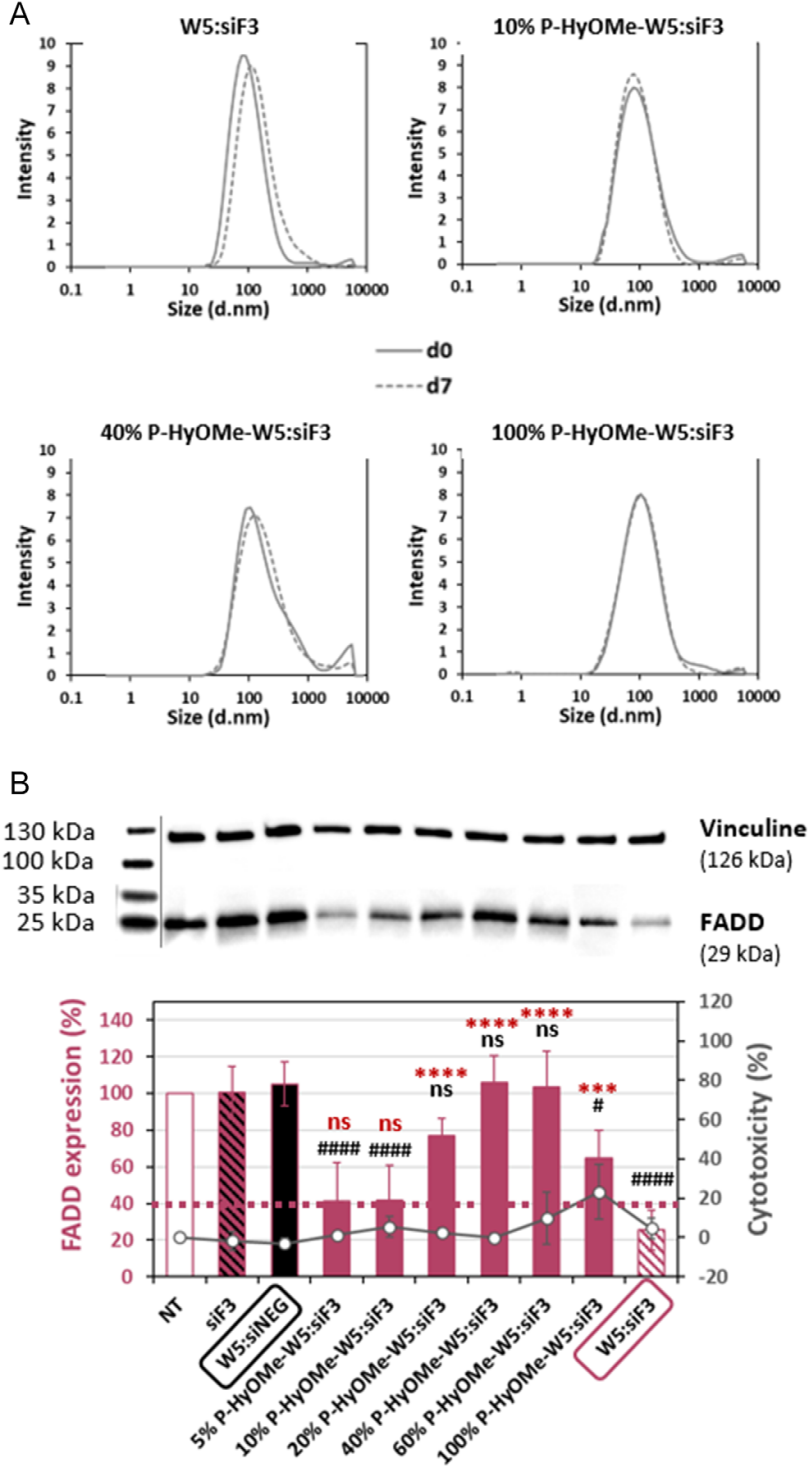
Stability and activity of PEG‐HyOMe‐WRAP5:siF3 nanoparticles with different percentages of PEGylation. A) Graphical representation of nanoparticle mean size with different percentages of P‐HyOMe‐W5 (*R* = 20, with siRNA = 500 nM). B) Graphical representation of FADD expression in EA.hy926 cells treated with nanoparticles detected by Western blot. Data represent mean ± SD, with *n* = 3 independent experiments in duplicates. Conditions: Nanoparticles with *R* = 20, siRNA = 40 nM/negative control: W5:siNEG (siRNA without a target) or the siF3 alone/Dashed line indicated 40% of FADD expression and the tolerated threshold of 20% cytotoxicity. Statistical analysis: One‐way ANOVA with Turkey post‐test, ns > 0.05, ** = 0.0002, and **** < 0.0001 versus W5:siF3 (pink square), or ns > 0.05, # = 0.0491, and #### < 0.0001 versus W5:siNEG (black square).

Even if the impact of P‐HyOMe conjugate on nanoparticle property is not fully understood, we investigated whether a reduced PEG‐HyOMe percentage could increase the nanoparticle activity. For that purpose, P‐HyOMe‐W5 and W5 peptide mixtures (5%, 10%, 20%, 40%, and 60% P‐HyOMe‐W5) were added to the siF3, and the nanoparticle formation was analyzed by DLS (**Figure** **4**A and Table S4, Supporting Information). Compared to naked W5:siF3% and 100% P‐HyOMe‐W5:siF3, we observed similar nanoparticle sizes and PdIs for 5% and 60% P‐HyOMe‐W5 (≈95 nm, 0.3), whereas the condition with 40% P‐HyOMe‐W5 seemed to be more labile, showing a bigger size (>130 nm) and higher PdI (>0.4). Curiously, we revealed for 10% and 20% P‐HyOMe‐W5:siF3 smaller sizes (≈70 nm) and lower PdIs (<0.3). At this percentage of P‐HyOMe‐W5, it could be that the PEGylation stabilized the nanoparticle formation due to less steric hindrance of the PEG moiety.

Afterward, we evaluated the FADD‐silencing properties of these various nanoparticles on EA.hy926 cells incubated first in serum‐free medium for 90 min before supplementing the medium with 10% serum (Figure 4B). Compared to the naked W5:siF3 nanoparticles, we obtained a significant reduction of FADD expression with the 5% and 10% P‐HyOMe‐W5:siF3 nanoparticles (≈70%, ns for both vs. W5:siF3). The activity of the 10% P‐HyOMe‐W5:siF3 nanoparticles could be explained by a higher positive surface charge reflected by a ZP value of 30.0 ± 5.2 mm, similar to W5:siF3.

The condition with 20% P‐HyOMe‐W5 displayed the same intermedia silencing property as the 100% P‐HyOMe‐W5:siF3 nanoparticles (≈40%, ****/*** versus W5:siF3, but ns between both conditions).

In conclusion, two nanoparticle formulations (10% or 100% P‐HyOMe‐W5) were based on 1) the DLS measurements at 7 days postformulation (Table S3, Supporting Information); and 2) the FADD knockdown results (Figure 4) showing that the nanoparticles with 10% or 100% P‐HyOMe‐W5 were stable with different silencing in physiological conditions (pH 7). These two nanoparticle formulations were further evaluated compared to the naked WRAP5:siF3 nanoparticles for their activity at pH 5 reflecting the extracellular pH during AMI.

### 2.4 Efficient FADD Knockdown Using pH‐Sensitive PEG‐WRAP5 Nanoparticles in Endothelial Cells at pH 5

To analyze both pH‐sensitive PEG‐HyOMe‐WRAP5:siF3 nanoparticles and naked W5:siF3 in pathophysiological conditions, we performed the nanoparticle transfection at pH 5 in a cell culture medium without serum. First, we confirmed that the vascular endothelial EA.hy926 cells were viable under this strong condition and during the transfection period of at least 24 h. As shown in Figure S8, Supporting Information, the cells revealed no morphological changes at pH 5 when incubated for 48 h. However, to ensure a stable acidic pH during the incubation period, we used a buffered DMEM medium (DMEM‐F12 at pH 5) for the transfection.

**Figure 5:**
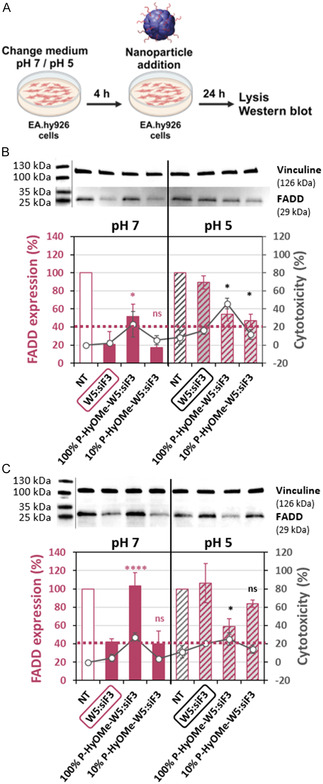
Comparison of FADD silencing under physiological and pathological conditions. A) Scheme of the nanoparticle transfection on human endothelial EA.hy926 cells at pH 7 and pH 5 (by BioRender). Graphical representation of FADD expression by Western blot of EA.hy926 cells treated (24 h) with W5:siF3, 100% P‐HyOME‐W5:siF3, and 10% P‐HyOMe‐W5:siF3 nanoparticles in a medium at pH 7 or pH 5 without B) serum or with C) 10 % serum. Data represent mean ± SD, with *n* = 3 independent experiments in duplicates. Transfection condition: Nanoparticles with *R* = 20, siRNA = 40 nm/dashed line indicated 40% of FADD expression. Statistical analysis: One‐way ANOVA with Turkey post‐test, ns > 0.05, * < 0.05, and **** < 0.0001 versus W5:siF3 with the respective color code.

Having ensured that the incubation condition at pH 5 did not significantly impact cell viability, we started to compare the FADD silencing using W5:siF3, 100% P‐HyOMe‐W5:siF3, or 10% P‐HyOMe‐W5:siF3 at pH 7 and pH 5 (**Figure** **5**). First, EA.hy926 cells were preincubated for 4 h with fresh media at pH 7 or pH 5 under serum starvation. Then, the nanoparticles were added for 90 min to the cells before supplementing the medium with 10% serum (pH 5 or 7). After 24 h, cell lysis and Western blot analysis were performed (Figure 5A).

In Figure 5B, we observed at pH 7 a FADD knockdown of ≈70% for W5:siF3% and 10% P‐HyOMe‐W5:siF3 compared to a FADD silencing of ≈40% for 100% P‐HyOMe‐W5:siF3 as previously observed (Figure 3B and 4B). At pH 5, the situation was different since we did not observe any FADD silencing using the naked W5:siF3 nanoparticles (Figure 5B). Interestingly, only both PEGylated versions induced a significant FADD knockdown of ≈50%. These results show that, under acidic conditions, the PEGylation of W5 nanoparticles was required for their stability and resulting activity.

In a second step, we evaluated FADD silencing with the corresponding nanoparticles in serum‐containing media during the entire incubation to avoid slightly increasing cytotoxicity at pH 5 (Figure 5B). At pH 7, we observed FADD silencing for the three nanoparticles comparable to that shown in the serum‐free medium, even if the knockdown efficiency is lower due to the presence of the serum (Figure 5C). At pH 5, we again obtained unexpected results: no FADD expression reduction was observed with W5:siF3 and 10% P‐HyOMe‐W5:siF3 nanoparticles. Only the 100% P‐HyOMe‐W5:siF3 nanoparticles induced a significant FADD silencing of 40% (* < 0.05 vs W5:siF3).

Thereafter, we analyzed FADD silencing induced by 100% P‐HyOMe‐W5:siF3 and W5:siF3 nanoparticles in a dose‐dependent manner (with 10% serum) at pH 5 on EA.hy926 cells (Figure S9, Supporting Information). For the W5:siF3 nanoparticles, we observed a dose‐dependent FADD silencing reaching 60% for 80 nM siF3. In contrast, for the 100% P‐HyOMe‐W5:siF3 nanoparticles, FADD knockdown was maximal (60%) for 40 nM siF3. FADD silencing did not increase with increasing siRNA concentration, probably due to the dose‐dependent increase in cytotoxicity.

Taken together, our results show that naked W5:siF3 nanoparticles are not an appropriate delivery system in acidic cellular conditions. Only the 100% P‐HyOMe‐W5:siF3 nanoparticles can induce FADD silencing under stress conditions associated with extracellular acidic pH 5 conditions, as observed during myocardial ischemia‐reperfusion injury. Moreover, this effect was conserved if the nanoparticle incubation was performed in the presence of serum.

### 2.5 Comparing SiRNA Internalization in Endothelial Cells at pH 7 versus pH 5

**Figure 6:**
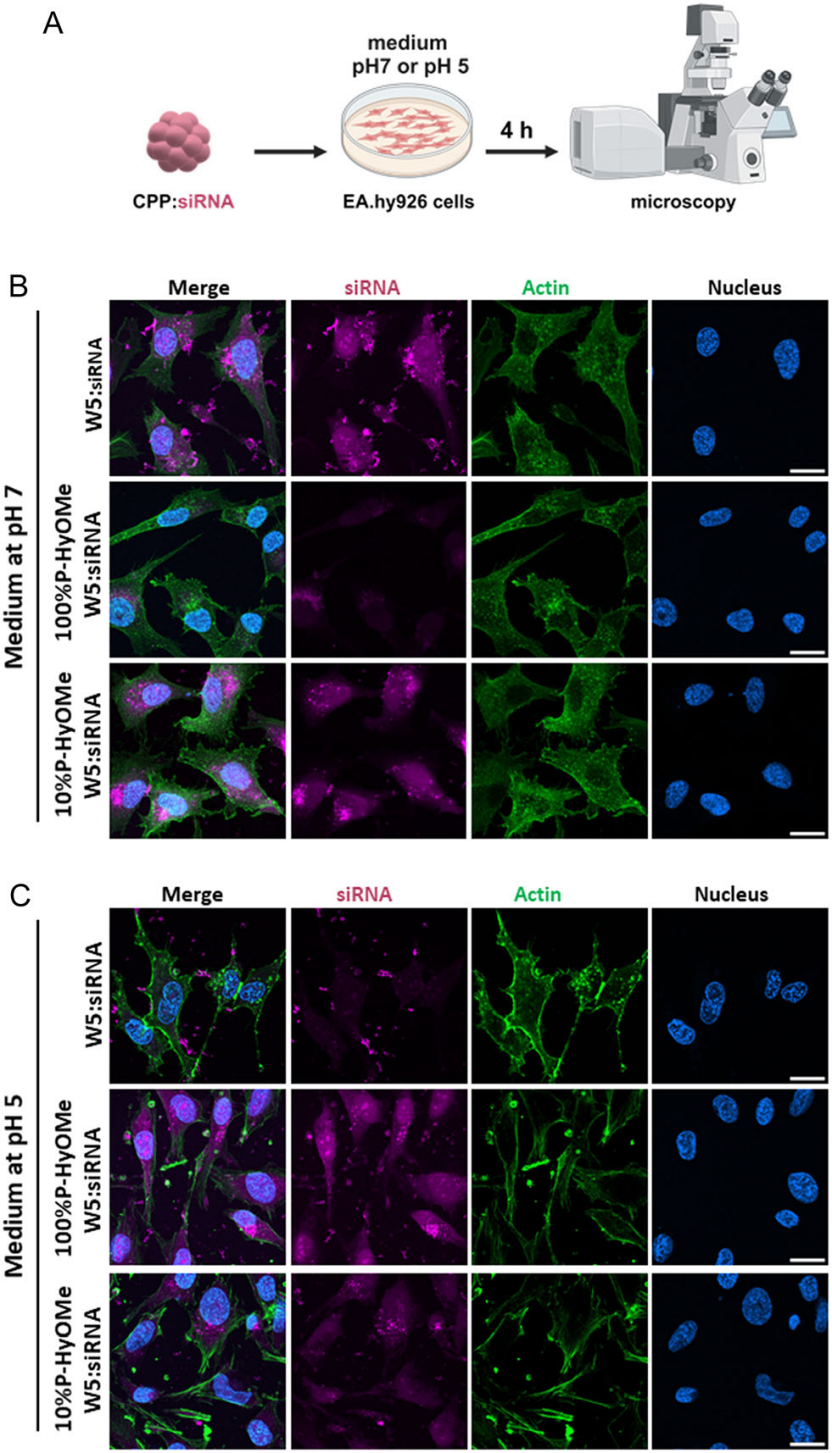
Visualization of the EA.hy926 cellular internalization of CPP:siF3 depending on the used pH. A) Scheme of the nanoparticle transfection on human vascular endothelial EA.hy926 cells at pH 7 and pH 5 (by BioRender). Representative confocal microscopy images were acquired of EA.hy926 cells incubated with nanoparticles as indicated (*R* = 20, siRNA‐Alexa546 = 40 nM) at B) pH 7 and at C) pH 5. Green = Phalloidin staining, magenta = siRNA‐Alexa546, blue = Hoechst nucleus staining. White bar = 10 μm.

To better understand the silencing activity of the different nanoparticles (Figure 5 and S9, Supporting Information), we encapsulated a fluorescence‐labeled siRNA using various formulations to follow its cellular internalization in EA.hy926 cells at pH 5 versus pH 7 by confocal microscopy (**Figure** **6**). Therefore, vascular endothelial cells were incubated with the nanoparticles for 4 h in a serum‐free medium at both pH before cell fixation (Figure 6A). At pH 7, we observed a significant signal of the fluorescent siRNA (magenta color in Figure 6B) in the cells transfected by the W5 peptide compared to nontreated cells (condition not shown). However, some aggregates were also visible outside the cells and at the cell membranes. In contrast, for the 10% P‐HyOMe‐W5:siRNA‐Alexa546 nanoparticles, the intracellular signal was comparable to that observed for W5:siRNA‐Alexa54, but without the aggregations, meaning that the PEGylation seemed to stabilize the nanoparticles in the culture medium. For the 100% P‐HyOMe‐W5:siRNA‐Alexa546 nanoparticles, the cellular internalization was drastically reduced in agreement with their lower FADD‐silencing ability (≈40% vs ≈70% for the others) as quantified by Western blot analysis (Figure 3B and 4B).

At pH 5 (Figure 6C), we had nearly no cellular internalization of the W5:siRNA‐Alexa546 nanoparticles, but some aggregations around the cells confirmed the missing FADD‐silencing activity under this condition. A higher cellular internalization was determined for 10% P‐HyOMe‐W5:siRNA‐Alexa546 nanoparticles with fewer extracellular aggregations coherent with the observed FADD silencing of ≈50% (Figure 5B). Finally, the highest cellular internalization was observed at pH 5 for the 100% P‐HyOMe‐W5:siRNA‐Alexa546 nanoparticles in agreement with an adequate FADD knockdown observed in acidic conditions (Figure 5B,C).

In order to ascertain the behavior of the nanoparticles in acidic conditions, we conducted additional DLS measurements. First, we determined the mean sizes and PdI values of W5:siF3 and 100%P‐HyMOe‐W5:siF3 nanoparticles under standard formulation conditions. Subsequently, we added 5 μL HCl (0.1N) to the formulation to reduce the pH to 5 and then measured the solutions again. The results showed that the naked W5 nanoparticles aggregated under acidic conditions, whereas the PEGylated version stayed unchanged (see Table S5, Supporting Information). Once the PEG moiety was cleaved from the nanoparticles under acidic conditions, the resulting nanoparticles corresponded to that formulated with the Ald‐W5 peptide. Evaluation of the Ald‐W5:siF3 nanoparticles by DLS under acidic conditions revealed that the remaining aldehyde (see Scheme 1) is sufficient to keep the nanoparticles stable at pH 5.

The findings indicated a direct correlation between FADD silencing activity and the stability of naked versus PEGylated pH‐sensitive WRAP5 nanoparticles depending on the extracellular pH environment. It is imperative that the nanoparticles exhibit stability within the specific cell environment to ensure optimal internalization and silencing activity.

### 2.6 FADD Knockdown in Human Cardiomyocytes Using PEGylated pH‐Sensitive WRAP5 Nanoparticles at pH 5

Given the higher number of cardiomyocytes compared to endothelial cells in the human heart, our interest lies in evaluating the PEGylated pH‐sensitive WRAP5 nanoparticles in cardiomyocytes since both vasculo‐ and cardioprotection are required to save the cardiac muscle during reperfusion injury.

Because human cardiomyocytes are unavailable as cell lines, we conducted the proof of concept using rat H9c2 cardiomyocytes. We utilized specific siRNA (siFr1 and siFr3, sequences provided in Table S1, Supporting Information) to knock down FADD protein expressed in rat cells. W5:siFr3 nanoparticles (siRNA = 80 nM) resulted in ≈50% silencing of the rat FADD protein (Figure S10, Supporting Information) confirming the silencing FADD protein in cardiomyocytes.

**Figure 7:**
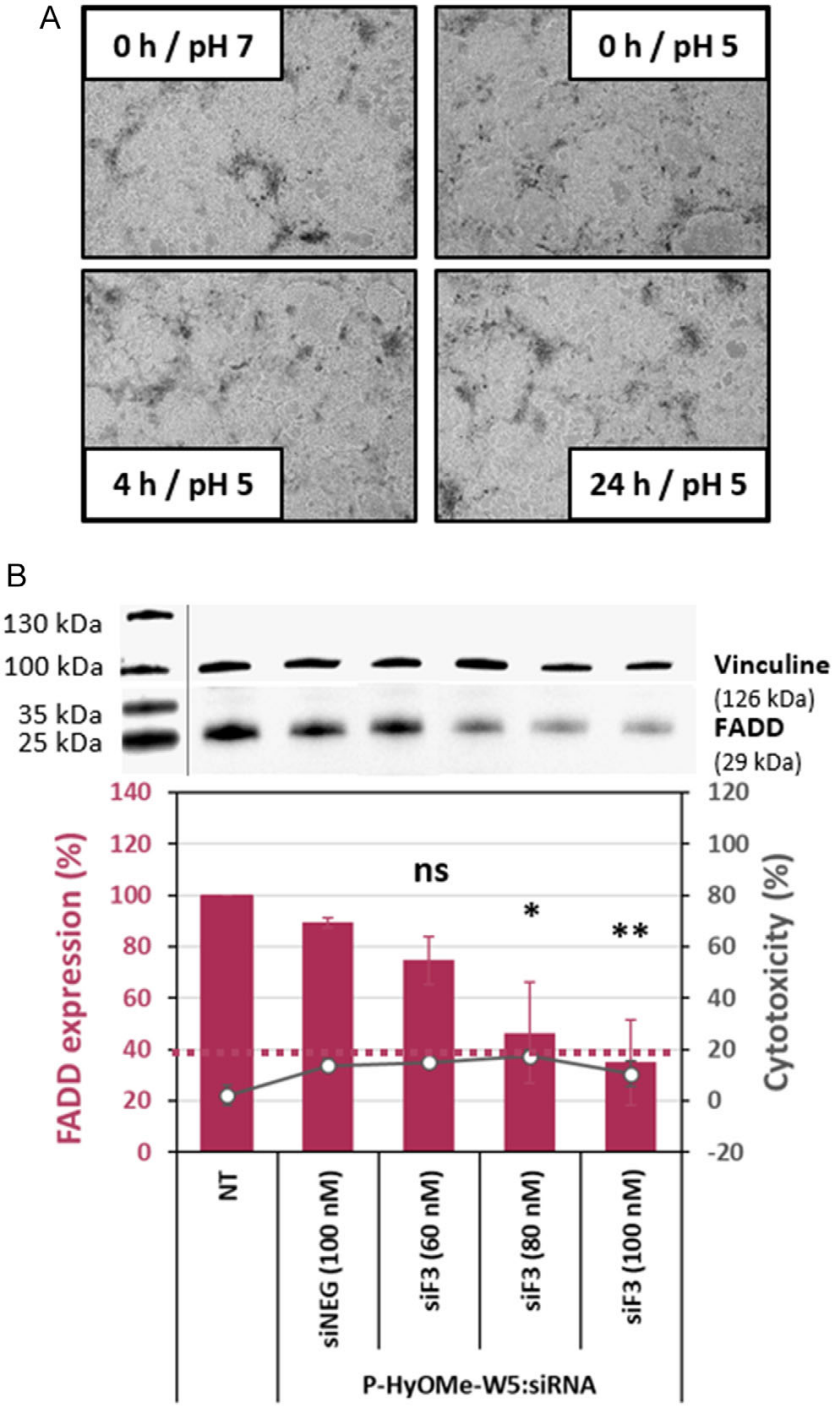
FADD silencing in hiPSC‐derived cardiomyocytes. A) Representative images of hiPSC‐derived cardiomyocytes incubated in media at different pH values confirm no effect due to pH changes. B) hiPSC‐derived cardiomyocytes were incubated 100% P‐HyOME‐W5:siF3 in a dose‐dependent manner in a medium at pH 5 without serum for 24 h. Western blot‐monitored FADD expression. Data represent mean ± SD, with *n* = 2 independent experiments. Conditions: Nanoparticles with *R* = 20, siF3 between 60 and 100 nM/dashed line indicated 40% of FADD expression, and the tolerated threshold of 20% cytotoxicity. Statistical analysis: One‐way ANOVA with Turkey post‐test, ns > 0.05, * < 0.05, and ** < 0.01 versus P‐HyOMe‐W5:siNEG.

Later on, we assessed the impact of FADD silencing in a more physiological setting, in beating hiPSC‐derived cardiomyocytes^[^ ^45^ ^]^ (refer to Video S1, Supporting Information). First, we confirmed that incubating hiPSC‐derived cardiomyocytes at pH 5 for 24 h did not alter cell morphology (**Figure** **7**A). Then, we exposed them to 100% P‐HyOMe‐W5:siF3 while increasing the siRNA concentrations from 60 to 100 nM and compared the FADD silencing to that achieved with 100% P‐HyOMe‐W5:siNEG (100 nM) (Figure 7B). The siF3 concentrations were increased for hiPSC‐derived cardiomyocytes compared to EA.hy926 due to the higher cell number in each culture well to keep approximatively the same nanoparticle‐to‐cell ratio.

At the highest siF3 concentration of 100 nM, we observed a 60% reduction in FADD expression in the confluent CMs, which was not seen in untreated CMs or those treated with 100% P‐HyOMe‐W5:siNEG.

More importantly, as observed by microscopy after treatment, 100% P‐HyOMe‐W5:siRNA incubation of the hiPSC‐derived CM did not induce specific cytotoxicity (values below the threshold of 20%) (Figure 7B)

This result emphasizes that the delivery system, consisting of 100% P‐HyOMe‐W5, could specifically target cardiomyocytes and endothelial cells when a pH drop occurs, such as during myocardial ischemia, and deliver siRNA as a therapeutic tool to treat reperfusion injury.

## 3 Conclusion

Our study provides a therapeutic tool formulated with WRAP5‐based nanoparticles able to specifically inactivate the expression of FADD, a protein known to mediate apoptotic cascades during ischemia‐reperfusion injury. However, during the ischemic period an extracellular pH drop to 5 occurs, which could influence siRNA delivery. Indeed, we could observe that naked WRAP5 nanoparticles are totally inefficient under acidic conditions in human vascular endothelial cells. Therefore, we have developed PEGylated WRAP5 nanoparticles with a finely tuned pH‐sensitive acyl hydrazone linker. The PEG‐HyOMe‐WRAP5 nanoparticles were able to encapsulate FADD‐targeting siRNA to form stable nanoparticles of 103.6 ± 16.2 nm mean size with a PdI of 0.301 ± 0.064, similar to the naked WRAP5 nanoparticles. More importantly, only this upgraded version internalized and induced FADD silencing at pH 5 in human vascular endothelial EA.hy926 cells or hiPSC‐derived cardiomyocytes.

In the acidic extracellular environment, PEG moieties are cleaved, liberating the positive surface charges of the nanoparticles required for cellular internalization. Our pH‐sensitive mechanism is drastically different from that of pH‐sensitive ionized lipid nanoparticles, which are activated in the endosome once the nanoparticle is internalized.

In conclusion, targeting two important cell types of the human heart is a promising result for further ex vivo or in vivo studies. In addition, PEGylation of the WRAP5 nanoparticles could be important for an in vivo administration (intravenous or intracardiac) as the PEG moiety can stabilize the nanoparticles by masking the interactions with the serum proteins.^[^ ^46^, ^47^ ^]^

## 4 Experimental Section

Details on materials and methods are given in the Supporting Information.

## Supporting Information

The authors have included the whole Experimental Section, Tables S1 to S3, and Figures S1 to S10 in the Supporting Information. They have cited additional references within the Supporting Information.^[^ ^42^ ^]^

## Conflict of Interest

The authors declare no conflict of interest.

## Supporting information

Supplementary Material

## Data Availability

The data (protocols, peptide characterizations, movie, etc.) that support the findings of this study are available in the supplementary material of this article.
