# Supplementary material for: Enhancing WRAP‐Based Nanoparticles for Small Interfering Ribonucleic Acid Delivery in pH‐Sensitive Environments
Source: ChemMedChem. 2025 Apr 10;20(11):e202400885. doi: 10.1002/cmdc.202400885 (PMC12132915; doi:10.1002/cmdc.202400885)
Supplement: Supplementary file 1 — Supplementary Material [file CMDC-20-e202400885-s001.zip › pHsensitive_WRAP_Supplement_revised_vf.pdf]

Enhancing WRAP-based Nanoparticles for siRNA Delivery in pH-Sensitive Environments

Giulia Di Gregorio[a]+, Coélio Vallée[b]+, Karidia Konate[a], Clémentine Teko-Agbo[a], Thania Hammoum[a], Héloïse Faure-Gautron [a], Yannick Bessin[b], Sebastien Deshayes[a], Eric Vivès[a], Albano C Meli[a], Pascal de Santa Barbara[a], Sandrine Faure[a], Stéphanie Barrère-Lemaire[c], Sébastien Ulrich*[b], Prisca Boisguérin*[a]

[a] Giulia Di Gregorio, Dr. Karidia Konate, Clémentine Teko-Agbo, Thania Hammoum, Héloïse Faure-Gautron, Dr. Sebastien Deshayes, Dr. Eric Vivès, Dr. Albano C Meli, Dr. Pascal de Santa Barbara, Dr. Sandrine Faure, Dr. Prisca Boisguérin
PhyMedExp,
University of Montpellier, INSERM, CNRS,
371 Av. Doyen Giraud, 34295 Montpellier, France
E-mail: prisca.boisguerin@inserm.fr

[b] Coélio Vallée, Dr. Yannick Bessin, Dr. Sébastien Ulrich
IBMM, Institut des Biomolécules Max Mousseron,
Université de Montpellier, CNRS, ENSCM
1919 route de Mende, 34293 Montpellier, France
E-mail: sebastien.ulrich@cnrs.fr

[c] Dr. Stéphanie Barrère-Lemaire
Institut de Génomique Fonctionnelle,
University of Montpellier, CNRS, INSERM CNRS
141 rue de la Cardonille, 34094 Montpellier, France

[+] These authors contributed equally to this work.

SUPPLEMENT MATERIAL

MATERIALS AND METHODS

All reagents and solvents were obtained from commercial sources and were used without further purification. The mPEG2000-hydrazide was purchased from Biopharma PEG.

High-performance liquid chromatography (HPLC).
Analytical HPLC was performed on a Thermo Scientific, UltiMate3000 equipped with a C18 reversed-phase column (Thermo Scientific, hypersilGOLD aQ, 2.1 x 50 mm, 1.9 µm). Elution was performed using a binary gradient (solvent A: H_2O + 0.1% TFA; solvent B: ACN + 0.1% TFA). *Method A*: 0 to 95% of solvent B in 5 min then 2 min at 95% of solvent B; flow: 0.5 mL/min; *Method B:* 20 to 70% of solvent B in 30 min; flow: 0.5 mL/min. Retention times (t_R) are given in minutes.
Preparative HPLC was performed using a binary gradient elution on a GILSON PLC2250, at semi-preparative scale (NUCLEODur Semi-prep, C18 HTEC, 7µm, (250 x 21 mm) column, Macherey-Nagel; flow 25mL/min), or preparative scale (XSelect CSH Prep C18, 5 µm, (250 × 30 mm) column, Waters; flow 45 mL/min). HPLC eluents: (Solution A: 99.9% Water, 0.1% TFA; Solution B: 99.9% Acetonitrile, 0.1% TFA; Solution C: 100% Water, no TFA; Solution D: 100% Acetonitrile, no TFA).

Liquid chromatography-mass spectrometry (LC/MS).
Analyses were performed on a Shimadzu LCMS2020 (Phenomex Kenetex C18, 2.6 µm ^{x} 7.5 cm, 100Å) equipped with a SPD-M20A detector with the following linear gradient of solvent B (99.9% acetonitrile, 0.1% HCOOH) and solvent A (99.9% water and 0.1% HCOOH): 5 to 95% of solvent B in 5 min; flow 1 ml/min. Retention times (t_R) are given in minutes.

Mass spectrometry (MS). Matrix Assisted Laser Desorption Ionization – Time Of Flight (MALDI-TOF) analyses were carried out at the Laboratoire de Mesures Physiques, IBMM, Université de Montpellier using Micromass Q-Tof instruments.

Nuclear magnetic resonance spectroscopy (NMR). ^{1}H NMR spectra were recorded at 400 MHz on Bruker Avance 400 or ARX instruments in deuterated solvents. Peaks were referenced in ppm to the residual solvent peak. Data are reported as follows: chemical shift (δ in ppm), multiplicity (s for singlet, d for doublet, t for triplet, m for multiplet), coupling constant (*J* in Hertz), and integration.

Synthesis and characterization of model mPEG2000 acyl hydrazone conjugates
These compounds were obtained through the reaction of benzaldehyde derivatives with mPEG2000-hydrazide (5 eq.) in EtOH/AcOH (50/50; 50 mM in benzaldehyde derivatives) at 78°C for 2h. The crude products were concentrated in a vacuum, then diluted with H_2O/ACN and purified by semi-preparative HPLC with gradients C/D (5% of solvent D for 5 min, then 5% to 100% of solvent D in 45 min and 100% solvent D for 5 min) to afford the pure products.

PEG-H. Analytical HPLC t_R: 4.337 min (*Method A*); ^{1}H NMR (400 MHz, DMSO-d_6): δ 3.24 (s, 3H, CH_3), 3.41-3.69 (m, 180H, CH_2CH$_2$), 4.22 (t, *J* = 4.3, 2H, CH_2CO), 7.40 (m, 3H, H_{arom}), 7.62 (dd, *J* = 8.0, 1.6, 2H, H_{arom}), 8.02 (s, 1H, C*H*).
PEG-F. Analytical HPLC t_R: 4.393 min (*Method A*)**;** ^{1}H NMR (400 MHz, DMSO-d_6): δ 3.24 (s, 3H, CH_3), 3.41 – 3.69 (m, 194H, CH_2CH$_2$), 4.23 (t, *J* = 4.4, 2H, CH_2CO), 7.25 (m, 2H, H_{arom}), 7.44 (m, 1H, H_{arom}), 7.83 (t, *J* = 7.48, 1H, H_{arom}), 8.25 (s, 1H, C*H*).
PEG-Cl. Analytical HPLC t_R: 4.467 min. (*Method A*); ^{1}H NMR shift (400 MHz, DMSO-d_6): δ 3.24 (s, 3H, CH_3), 3.51 (s, 179H, CH_2CH$_2$), 4.23 (t, *J* = 4.4, 2H, CH_2CO), 7.40 (m, 2H, H_{arom}), 7.49 (m, 1H, H_{arom}), 7.90 (m, 1H, H_{arom}), 8.43 (s, 1H, C*H*).

PEG-Br. Analytical HPLC t_R: 4.493 min (*Method A*); ^{1}H NMR (400 MHz, DMSO-d_6): δ 3.24 (s, 3H, C*H*$_3$), 3.41 – 3.69 (m, 182H, C*H*$_2$CH$_2$), 4.23 (t, *J* = 4.4 Hz, 2H, CH$_2$CO), 7.33 (td, *J* = 7.9, 1.8, 1H, *H*$_{arom}$), 7.43 (t, *J* = 7.4, 1H, *H*$_{arom}$), 7.66 (dd, *J* = 8.0, 1.0, 1H, *H*$_{arom}$), 7.88 (dd, *J* = 7.8, 1.6, 1H, *H*$_{arom}$), 8.39 (s, 1H, C*H*).
PEG-OH. Analytical HPLC t_R: 4.380 min (*Method A*); ^{1}H NMR (400 MHz, DMSO-d_6): δ 3.24 (s, 3H, C*H*$_3$), 3.41 – 3.69 (m, 180H, C*H*$_2$CH$_2$), 4.23 (t, *J* = 4.4, 2H, C*H*$_2$CO), 6.87 (t, *J* = 8.2, 2H, *H*$_{arom}$), 7.25 (dt, *J* = 7.8, 1.6, 1H, *H*$_{arom}$), 7.46 (dd, *J* = 7.6, 1.6, 1H, *H*$_{arom}$), 8.23 (s, 1H, C*H*), 10.84 (s, 1H, O*H*).
PEG-OMe. Analytical HPLC t_R: 4.390 min (*Method A*); ^{1}H NMR shift (400 MHz, DMSO-d_6): δ 3.25 (s, 3H, C*H*$_3$), 3.41 – 3.69 (m, 199H, C*H*$_2$CH$_2$), 3.83 (s, 3H, C*H*$_3$), 4.21 (t, *J* = 4,1, 2H, C*H*$_2$CO), 6.98 (t, *J* = 7.5, 1H, *H*$_{arom}$), 7.07 (d, *J* = 8.3, 1H, *H*$_{arom}$), 7.38 (td, *J* = 8.9, 1.8, 1H, *H*$_{arom}$), 7.75 (dd, *J* = 7.8, 1.4, 1H, *H*$_{arom}$), 8.37 (s, 1H, C*H*).

Solid Phase Peptide Synthesis (SPPS)

The WRAP5 peptide *(Leu-Leu-Arg-Leu-Leu-Arg-Trp-Trp-Trp-Arg-Leu-Leu-Arg-Leu-Leu)* was synthesized by solid-phase peptide synthesis, as previously reported using a standard Fmoc approach,[2-4] at the SynBio3 platform, Institut des Biomolécules Max Mousseron, on a Liberty BlueTM Microwave Peptide Synthesizer (CEM Corporation, Matthews, NC, USA).
The synthesis was conducted on a 0.1 mmol scale on a Rink amide resin. The Fmoc deprotection was performed with a 20% piperidine in N,N-Dimethylformamide (DMF) solution. All coupling reactions were performed with 5 eq. of Oxyma in DMF (0.5 M, 1 mL), 5 eq. of amino acids in DMF (0.2 M, 2.5 mL), and 10 eq. of DIC in DMF solution (1 M, 1 mL) for 2 min at 90 °C. Each deprotection and coupling reaction was performed at 90 °C under nitrogen bubbling. Each cycle was characterized by two deprotection steps for 1 min at 90 °C, three washings of 5 mL DMF, followed by a double coupling step (2 min) at 90 °C.
The subsequent functionalization of the WRAP5 peptide to graft the aldehyde moiety at the N-terminus was performed directly in the solid phase at a 0.1 mmol scale using benzoic acid derivatives (5 eq., 0.2 M in DMF), HATU (5 eq., 0.2 M in DMF) and DIEA (10 eq.). The coupling was left to react overnight. The cleavage and final deprotection of the functionalized WRAP5 peptide were done using a TFA/H_2O (95/5) deprotection cocktail during 3h30. Then, the solution was filtered out and the resin was washed with DCM. The cleavage cocktail and the DCM were gathered, concentrated under a vacuum, then retaken with H_2O/ACN (5/6, 11 mL) and heated to 95°C for 10 minutes before RP-HPLC purification.
Ald-W5. Synthesized according to the general protocol using 4-formylbenzoic acid. Purified by preparative HPLC (gradient: 20% B, 5 min, then 20% B to 80% B in 50 min, then 100% B for 5 min) to afford 45 mg (20% overall yield) of the desired product. Analytical HPLC t_R: 5.943 min (*Method A)*; ESI-MS m/z calcd for [$C_{113}H_{173}N_{31}O_{17}$+2H$^+$] 1119.18, found 1119.95; [$C_{113}H_{173}N_{31}O_{17}$+3H$^+$] 746.45, found 747.05; [$C_{113}H_{173}N_{31}O_{17}$+4H$^+$] 560.08, found 560.40.
OMe-Ald-W5. Synthesized according to the general protocol using 3-methoxy-4-formylbenzoic acid. Purification by preparative HPLC (gradient: 20% B, 5 min, then 20% B to 80% B in 50 min, then 100% B for 5 min) to afford 22 mg (10% overall yield) of the desired product. Analytical HPLC t_R: 6.010 min (*Method A*); ESI-MS m/z calcd for [$C_{114}H_{175}N_{31}O_{18}$+2H$^+$] 1134.69, found 1134.75; [$C_{114}H_{175}N_{31}O_{18}$+3H$^+$] 756.80, found 756.90; [$C_{114}H_{175}N_{31}O_{18}$+4H$^+$] 567.85, found 567.90.
AcHy-W5. Synthesized through the reaction of Ald-W5 (17 mg, 7.60 µmol) with acetic hydrazide (5 mg, 9 eq.) in EtOH/AcOH (50/50, 50 mM in Ald-W5, V_{tot} = 89.4 µL) at 78°C for 2h. The crude product was concentrated using a vacuum and then diluted in ACN/H_2O and purified by semi-preparative HPLC (gradient: 5% of B for 5 min then 5 to 100% of B in 50 min) to afford 9 mg (52% overall yield) of AcHy-W5. Analytical HPLC t_R: 5.893 min (*Method A*); ESI-MS m/z calcd for [$C_{115}H_{177}N_{33}O_{17}$+2H$^+$] 1147.71, found 1147.60; [$C_{115}H_{177}N_{33}O_{17}$+3H$^+$] 765.47, found 765.40; [$C_{115}H_{177}N_{33}O_{17}$+TFA+2H$^+$] 803.14, found 803.35; [$C_{115}H_{177}N_{33}O_{17}$+4H$^+$] 574.35, found 574.30.
AcHyOMe-W5. Synthesized through the reaction of **OMe-Ald-W5** (10 mg; 4.41 µmol) with acetic hydrazide (6.53 mg, 20 eq.) in EtOH/AcOH (50/50, 50 mM in **OMe-Ald-W5**, V_{tot} = 88 µL) at 78°C for 2 hours. The crude product was concentrated using a vacuum, and then diluted in ACN/H_2O and purified by semi-preparative HPLC to afford the desired product (2 mg, 20% yield). Analytical HPLC t_R: 5.89 min (*Method A*); ESI-MS m/z calcd for [$C_{116}H_{179}N_{33}O_{18}$+3H$^+$] 775.48, found 775.49; [$C_{116}H_{179}N_{33}O_{18}$+2TFA+2H$^+$] 1276.71, found 1276.72; [$C_{116}H_{179}N_{33}O_{18}$+4H$^+$] 581.86, found 581.87.
P-Hy-W5. Synthesized through the reaction of **Ald-W5** (16 mg, 7.15 µmol) with mPEG2000-hydrazide (29.2 mg, 2 eq.) in EtOH/AcOH (50/50, 50 mM in **Ald-W5**, V_{tot} = 143 µL) at 78°C overnight. The crude

product was concentrated using a vacuum and then diluted in ACN/H_2O and purified by semi-preparative HPLC (gradient: 5% of B for 5 min then 5 to 100% of B in 50 min) to afford 11 mg (35% overall yield) of **P-Hy-W5**. HPLC t_R: 5.770 min (*Method: A*).

P-HyOMe-W5. Synthesized through the reaction of **OMe-Ald-W5** (12 mg, 5.29 µmol) with mPEG2000-hydrazide (21.6 mg, 2 eq.) in EtOH/AcOH (50/50, 50 mM in **OMe-Ald-W5**, V_{tot} = 106 µL) at 78°C overnight. The crude product was concentrated using a vacuum and then diluted in ACN/H_2O and purified by semi-preparative HPLC (gradient: 5% of B for 5 min then 5 to 100% of B in 50min) to afford 10 mg (44% overall yield) of **P-HyOMe-W5**. HPLC t_R: 5.820 min (*Method: A*); MALDI-TOF (DHB), monoisotopic mass calcd for 45 ethylene glycol units: [$C_{207}H_{362}N_{33}O_{64}$+$H^+$] 4334.60, found 4334.50; 44 ethylene glycol units: [$C_{205}H_{358}N_{33}O_{63}$+$H^+$] 4290.58, found 4290.50; 43 ethylene glycol units: [$C_{203}H_{353}N_{33}O_{62}$+$H^+$] 4246.56, found 4246.50; 40 ethylene glycol units: [$C_{197}H_{341}N_{33}O_{59}$+$H^+$] 4114.48, found 4114.40; 35 ethylene glycol units: [$C_{187}H_{322}N_{33}O_{54}$+$H^+$] 3894.35, found 3894.30; 28 ethylene glycol units: [$C_{173}H_{294}N_{33}O_{47}$+$H^+$] 3586.16, found 3586.1.

Hydrolytic studies.

The different buffers were prepared as follows: **Buffer pH 8** (sodium phosphate, 20 mM): 610 mg of Na_2HPO_4 + 109 mg of NaH_2PO_4 in 250 mL of H_2O; **Buffer pH 7** (sodium phosphate, 20 mM): 439 mg of Na_2HPO_4 + 297 mg of NaH_2PO_4 in 250 mL of H_2O; **Buffer pH 6** (sodium acetate, 20 mM): 388 mg NaOAc + 15 µL AcOH in 250 mL of H_2O; **Buffer pH 5** (sodium acetate, 20 mM): 266 mg NaOAc + 103 µL AcOH in 250 mL of H_2O; Buffer pH4 (sodium acetate, 20mM): 62 mg NaOAc + 244 µL AcOH in 250 mL of H_2O. Each buffer was adjusted to the pH of interest using a solution of NaOH 10 M or HCl 1 M.

The kinetics studies were carried out by placing the acylhydrazone conjugate in 1 mL of buffer in a 1 cm cuvette, monitoring the absorption band at 304 nm (1 point measured every 15 min) on a Safas UVmc2 spectrophotometer. The data were then normalized using the following equation:

$$\frac{\mathrm{A} - A_{min}}{A_0 - A_{min}} \times 100$$

where A represents the measured absorbance of the sample, A_0 is the initial absorbance of the sample, and A_{min} is the minimal absorbance throughout the data set.

Nanoparticle formation

Stock solutions of WRAP5 peptides were prepared in pure water or acetonitrile (AcN) supplemented by pure water (20% AcN final concentration) or ethanol (EtOH) supplemented by pure water (20% EtOH final concentration). siRNA (Eurogentec) stock solutions were prepared in RNase-free water as recommended by the manufacturer. siRNA sequences used in this study are shown in Table S1.

Nanoparticles were formulated in 5% (m/v) glucose in pure water (Sigma-Aldrich) by adding first the peptide and then the siRNA at a molar ratio (R) of 20 (WRAP:siRNA = 20:1), followed by an incubation of at least 30 minutes at room temperature (or 0°C or 37°C, depending on the experiment). Formulated nanoparticles could be stored for several weeks at 4°C. Nanoparticles composed of different percentages of PEGylated WRAP5 were formulated as described above using a peptide mixture solution (WRAP5 + PEGylated WRAP5) at the indicated proportion.

Dynamic light scattering (DLS) and Zeta potential (ZP)

Nanoparticles (peptide = 20 µM, siRNA = 1 µM, R = 20) were characterized with a Zetasizer NanoZS (Malvern) in terms of mean size (Z-average) of the particles and homogeneity of their distribution (PDI).

Zeta potential was measured in 5% glucose with NaCl 1 mM. All results were obtained from at least two independent measurements, three runs for each measurement at 25°C.

Cell Culture conditions

EA.hy926 (human vascular endothelial cells) and H9c2 (rat cardiomyocytes) were grown in complete medium: DMEM containing L-glutamine (Life Technologies) supplemented with 10% fetal bovine serum (FBS, Sigma Aldrich) and 1% penicillin/streptomycin (Life Technologies).

hiPSC-derived cardiomyocytes were obtained hiPSC cell lines from healthy male control using monolayer protocol and sandwich approaches [44]. The hiPSC-CMs were maintained in RPMI medium supplemented with B27 (Gibco).

All cells were kept in a humidified incubator with 5% CO_2 at 37°C.

Cell transfection experiments

50,000 c/well EA.hy926 and H9c2 cells were seeded for the transfection at pH 7 and 75,000 c/well EA.hy926 were seeded for the transfection at pH 5 into a 24-well plate 24 h before the experiment. The corresponding medium as described above was adjusted to pH 5 using 1 M HCl.

The next day the growth medium was replaced by 200 µL of fresh warm serum-free DMEM at pH 7 or serum-free DMEM-F12 at pH 5. Then 50 µL of the nanoparticle solutions were added to the cells. 50 µL glucose (5%) was used for non-treated conditions (NT). After 1.5 h of incubation, 250 µL DMEM or DMEM-F12 supplemented with 20% FBS (final FBS concentration = 10%) was added to each well without withdrawing the transfecting agent. Cells were then incubated for another 24 h and finally lysed for FADD western blotting detection.

hiPSC-CMs in a 6-well plate were used at differentiation days between d16 and d24. On the day of the experiment, the medium was replaced by 1800 µL of fresh warm RPMI medium at pH 5. After 4 h, 200 µL of the nanoparticle solutions or 200 µL glucose (5%) were added to the cells. Cells were then incubated for another 24 h and finally lysed for FADD western blotting detection.

Cytotoxicity assay (LDH)

The potent cytotoxicity induced by the nanoparticles was evaluated using the Cytotoxicity Detection KitPlus (LDH, Roche Diagnostics). After the 24 h nanoparticle incubation in the 24-well plate (see cell transfection experiment above), at least one NT well was used as an LDH positive control (100% cytotoxicity) by adding Triton X-100 (Sigma-Aldrich) and incubating 15 minutes at 37°C (final concentration of 0.1%). 50 µL of the supernatant of each condition was transferred in a new clear 96-well plate (Greiner) in triplicates. 50 µL of the “dye solution/catalyst” mixtures were added to each well and the plate was then incubated for 30 min in the darkness at room temperature, as recommended by the manufacturer. 25 µL/well of HCl (1 N) were added to each well to stop the reaction before measuring the absorption at 490 nm (Infinite 200 Microplate Reader, Tecan). Relative toxicity (%) was calculated with the following formula: [(exp. value–value non-treated cells)/(value triton–value non-treated cells)] × 100.

Western blot

Cell lysis was performed using a RIPA buffer [50 mM Tris pH 8.0, 150 mM sodium chloride, 1% Triton X-100, 0.1% sodium dodecyl sulfate (SDS, Sigma-Aldrich), supplemented with protease inhibitors (0.1% v/v, SigmaFAST, Sigma-Aldrich)]. After the complete removal of the culture medium, cells were incubated with

130 µL/well of RIPA lysis buffer for 5 min on ice. Then, cells were scraped and transferred in a 1.5 mL tube. The lysates were centrifuged for 5 min, 13500 rpm at 4°C. Supernatants were collected and protein concentrations were determined using the Pierce BCA Protein Assay (ThermoFisher). Cell extracts were separated by electrophoresis on 4-20% Mini-PROTEAN® TGXTM Precast Gel (Bio-Rad). Samples were transferred onto Trans-Blot® Turbo™ Mini PVDF Transfer membrane (Bio-Rad). As antibodies, we used anti-human FADD (BD Transduction #610399), anti-mouse FADD (Abcam, #124812), anti-Vinculin rabbit mAb E1E9V (Cell Signaling, #13901), anti-mouse IgG HRP (Cell Signaling, #7076) and anti-rabbit IgG HRP (Cell Signaling, #7074). All antibodies were diluted corresponding as recommended by the manufacturer in 5% Bovin Albumin Serum (BSA, Thermo Scientific) in Tris Buffered Saline (TBS, pH 8) containing 0.05% Tween 20 (Sigma-Aldrich). Blots were revealed with the SuperSignal™ West Pico PLUS Chemiluminescent Substrate (ThermoFisher) on a Sapphire imager (Azure Biosystems). The signal intensities of the blots were quantified using ImageJ software. First, each FADD band was normalized to the corresponding Vinculin band. Then, each normalized value corresponding to a single condition was expressed as a percentage of the normalized value of non-treated cells (NT) (= 100%)using the following equation: FADD expression (%) = normalized value(condition x)/normalized value (NT) x 100.

Confocal Microscopy

For imaging, cells were seeded on glass coverslips. Before cell seeding (100,000 cells/mL), the coverslips were washed twice with D-PBS and transferred into a 12-well plate. After 24 h, cells were incubated with the nanoparticles (WRAP:siRNA-Alexa546, R = 20, 40nM in 1 mL of DMEM medium) for 4 h in a humidified incubator with 5% CO_2 at 37°C. For the non-treated cells (NT), 5% glucose was used instead of the nanoparticles. 10 min before the end of the incubation, Hoechst 33342 dye (Sigma-Aldrich) was added into each well for nucleus labeling. Then, cells were washed three times with D-PBS and fixed for 10 min with 2% paraformaldehyde (PFA, Sigma-Aldrich) at room temperature. Finally, the cells were washed with D-PBS three times and mounted with poly-(vinyl alcohol) Mowiol™ 4–88 glycerol Tris buffer (Biovalley) on glass slides for imaging.

Images were acquired using a C Pao 63x/1.2 W DIIIII lens on a Zeiss LSM 800 Confocal Laser Scanning Microscope. For all acquisition settings, the main excitation sources for confocal mode were a diode at 405 nm and a helium/neon laser at 561 nm and 640 nm. The parameters specific for each fluorophore were: Hoechst dye, excited at 405 nm, detected between 400 nm - 456 nm, Alexa546 excited at 561 nm, detected between 565 nm - 617 nm, and Alexa647 excited at 640 nm, detected between 656 nm – 700 nm. Image acquisition was done sequentially to minimize crosstalk between the fluorophores. Image J was used to merge and adjust with the same brightness and contrast parameters for each confocal image.

Statistical analysis

Statistical analysis was performed on the corresponding data set (mean ± SD) by applying a one-way ANOVA with Turkey post-test (GraphPad Prism version 8.0.2). Statistical results correspond to ns > 0.05, * <0.05, ** <0.01, *** <0.001, and **** <0.0001.

Reference

[44] Y. Sleiman, M. Souidi, R. Kumar, E. Yang, F. Jaffré, T. Zhou, A. Bernardin, S. Reiken, O. Cazorla, A. V. Kajava, A. Moreau, J.-L. Pasquié, A. R. Marks, B. B. Lerman, S. Chen, J. W. Cheung, T. Evans, A. Lacampagne, A. C. Meli, EBioMedicine 2020, 60, 103024.

Table S1: siRNA sequences used in this study

siRNA	ID	Sence (5' → 3')	Antisence (5' → 3')
**siFADD-1**	siF1	GCA-CUC-CUC-UUA-UUC-CUA-AdTdT	UUA-GGA-AUA-AGA-GGA-GUG-CdTdT
**siFADD-2**	siF2	GAA-GAC-CUG-UGU-GCA-GCA-UdTdT	AUG-CUG-CAC-ACA-GGU-CUU-CdTdT
**siFADD-3**	siF3	CUG-AGA-AUC-UGG-AAG-AAC-AdTdT	UGU-UCU-UCC-AGA-UUC-UCA-GdTdT
**siFADD-4**	siF4	GCA-UUU-AAC-GUC-AUA-UGU-GdTdT	CAC-AUA-UGA-CGU-UAA-AUG-CdTdT
**siFADD-R1**	siFr1	GCU-GGG-CAG-ACA-CGA-CCU-AdTdT	UAG-GUC-GUG-UCU-GCC-CAG-CdTdT
**siFADD-R3**	siFr3	GGU-GGA-AGA-AGC-AUU-GAU-GdTdT	CAU-CAA-UGC-UUC-UUC-CAC-CdTdT

Footnotes: siFADD-1/ -2/ -3 and -4 are siRNA targeting the human mRNA of the FADD protein. siFADD-R1 and -R3 are siRNA targeting the rat mRNA of the FADD protein.

Table S2: Comparison of nanoparticle size and PdI depending on the used solvent.

Conditions	Nanoparticles	Mean size (nm)	PdI
**H_2O**	W5:siF3	92.7 ± 19.4	0.275 ± 0.071
	Ald-W5:siF3	125.8 ± 46.4	0.469 ± 0.125
	Ac-W5:siF3	140.1 ± 56.5	0.347 ± 0.086
	P-Hy-W5:siF3	115.9 ± 73.5	0.361 + 0.081
	P-HyOMe-W5:siF3	95.6 ± 9.7	0.491 ± 0.074
**20% AcN**	W5:siF3	290.8 ± 91.9	0.458 ± 0.073
	Ald-W5:siF3	144.8 ± 11.1	0.415 ± 0.015
	Ac-W5:siF3	142.8 ± 49.4	0.341 ± 0.071
	P-Hy-W5:siF3	196.4 83.0	0.418 ± 0.093

Footnotes: Peptides were solubilized in water or 20% Acetonitrile. All CPP:siRNA complexes were formed at molar ratio 20, using a siRNA concentration of 500 nM in an aqueous solution of 5 % glucose. n ≥ 2 independent formulations (3 measures per run). n.d.: not determined, PdI: Polydispersity Index, AcN: Acetonitrile.

Table S3: Evaluation of the nanoparticle stability by DLS measurement

Nanoparticles	Day 0		Day 7	
	Size (nm)	PdI	Size (nm)	PdI
**W5:siF3**	85.7 ± 9.8	0.268 ± 0.034	104.0 ± 1.4	0.219 ±0.009
**Ald-W5:siF3**	86.6 ± 6.0	0.314 ± 0.045	n.d.	n.d.
**AcHy-W5:siF3**	115.5 ± 35.3	0.293 ± 0.055	n.d.	n.d.
**P-Hy-W5:siF3**	94.2 ± 10.8	0.273 + 0.043	n.d.	n.d.
**P-HyOMe-W5:siF3**	103.6 ± 16.2	0.301 ± 0.064	83.2 ± 0.4	0.282 ± 0.004
**W5:siF3**	143.5 ± 71.8	0.508 ± 0.236	317.2 ± 192.3	0.391 ± 0.144
**W5:siF3**	104.7 ± 19.8	0.335 ± 0.081	177.3 ± 14.6	0.284 ±0.076
**P-HyOMe-W5:siF3**	190.0 ± 82.4	0.596 ± 0.205	176.7 ± 29.3	0.441 ± 0.078
**P-HyOMe-W5:siF3**	102.0 ± 19.8	0.452 ± 0.098	137.7 ± 59.7	0.384 ± 0.112

Footnotes: Peptides were solubilized in water or 20% Ethanol. All CPP:siRNA complexes were formed at molar ratio 20, using a siRNA concentration of 500 nM in an aqueous solution of 5 % glucose. Formulated nanoparticles were stored at 4°C for 7 days before the DLS measurements. n ≥ 2 independent formulations (3 measures per run). n.d.: not determined, PdI: Polydispersity Index. Formulations in **black** were performed at room temperature, those in **red** at 37°C, and those in **blue** at 0°C.

Table S4: DLS characterization of WRAP5-based nanoparticles with different PEG percentages.

Nanoparticles	Day 0		Day 7	
	Size (nm)	PdI	Size (nm)	PdI
**W5:siF3**	85.7 ± 9.8	0.268 ± 0.034	104.0 ± 1.4	0.219 ±0.009
**5% P-HyOMe-W5:siF3**	97.2 ± 1.5	0.255 ± 0.019	137.8 ± 2.0	0.297 ± 0.060
**10% P-HyOMe-W5:siF3**	70.6 ± 6.6	0.302 ± 0.028	69.2 ± 9.2	0.252 ± 0.020
**20% P-HyOMe-W5:siF3**	75.9 ± 5.5	0.266 ± 0.013	75.7 ± 4.8	0.257 ± 0.017
**40% P-HyOMe-W5:siF3**	131.1 ± 12.1	0.438 ± 0.125	190.4 ± 92.6	0.442 ± 0.061
**60% P-HyOMe-W5:siF3**	104.7 ± 2.8	0.305 ±0.047	103.4 ± 4.2	0.279 ± 0.007
**100% P-HyOMe-W5:siF3**	100.1 ± 9.5	0.307 ± 0.049	83.6 ± 17.8	0.278 ±0.022

Footnotes: Peptides were solubilized in water or 20% Ethanol. All CPP:siRNA complexes were formed at molar ratio 20, using a siRNA concentration of 500 nM in an aqueous solution of 5 % glucose. n ≥ 2 independent formulations (3 measures per run). n.d.: not determined, PdI: Polydispersity Index.

Table S5: Comparison of nanoparticle size and PdI depending on the used solvent.

Nanoparticles	Conditions	Mean size (nm)	PdI
W5:siF3	normal	76.1 ± 6.2	0.300 ± 0.037
	+ HCl	1,320 ± 605	0.525 ± 0.049
P-HyOMe-W5:siF3	normal	87.4 ± 4.1	0.267 ± 0.010
	+ HCl	71.3 ± 6.3	0.464 ± 0.030
Ald-W5:siF3	normal	92.6 ± 2.0	0.261 ± 0.012
	+ HCl	88.9 ± 2.3	0.295 ± 0.052

Footnotes: Peptides were solubilized in water or 20% Ethanol. All CPP:siRNA complexes were formed at molar ratio 20, using a siRNA concentration of 500 nM in an aqueous solution of 5 % glucose for mean size and PdI acquisition corresponding to the “normal” condition. Then 5 µL 0.1 N HCl solution was added switching the pH to 5 to measure the influence on nanoparticle size and PdI (= + HCl condition). n ≥ 2 independent formulations (3 measures per run). n.d.: not determined, PdI: Polydispersity Index, can: Acetonitrile.

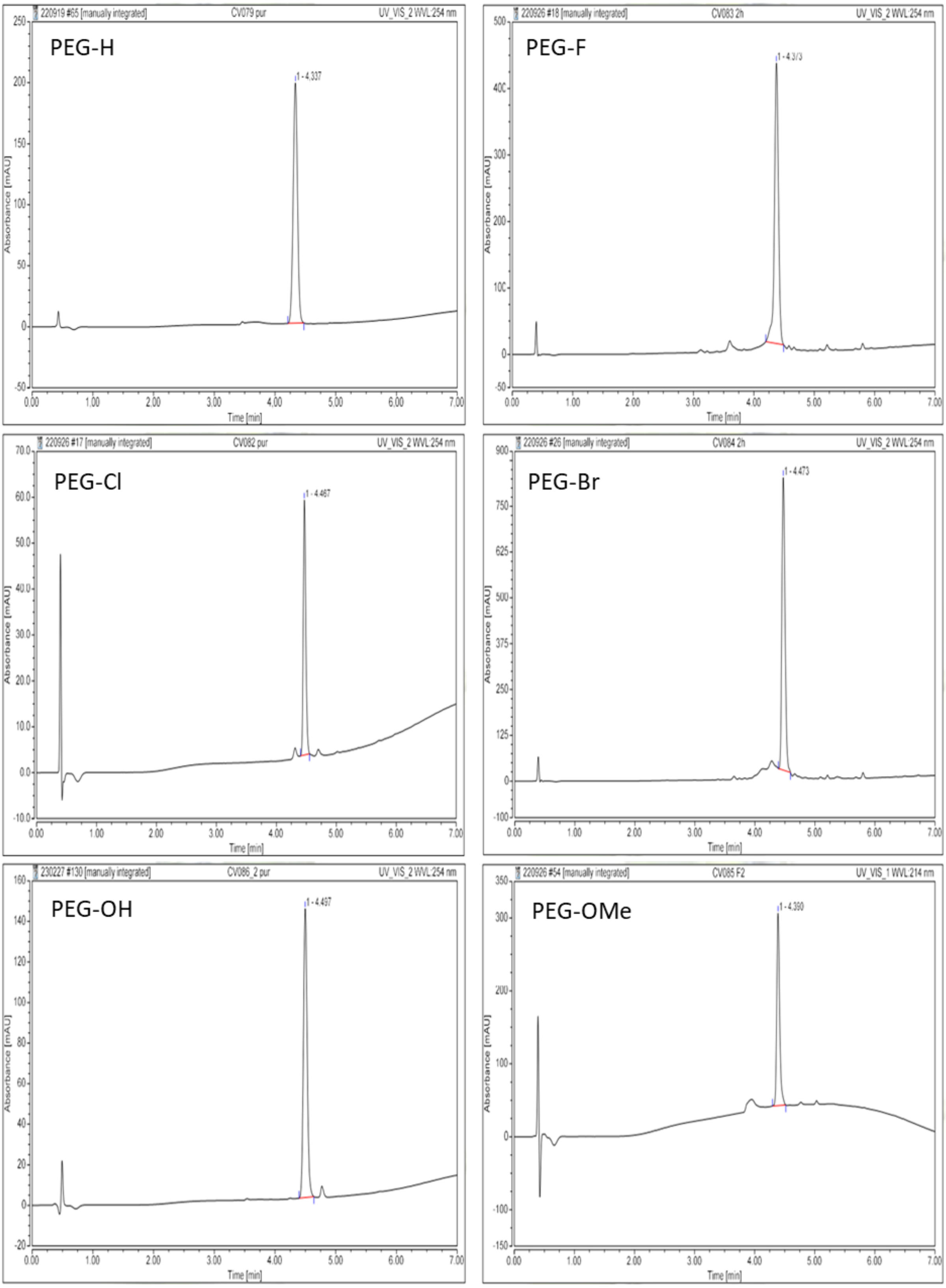

Figure S1: HPLC chromatograms of mPEG2000 acyl hydrazone model compounds featuring varying neighboring groups.

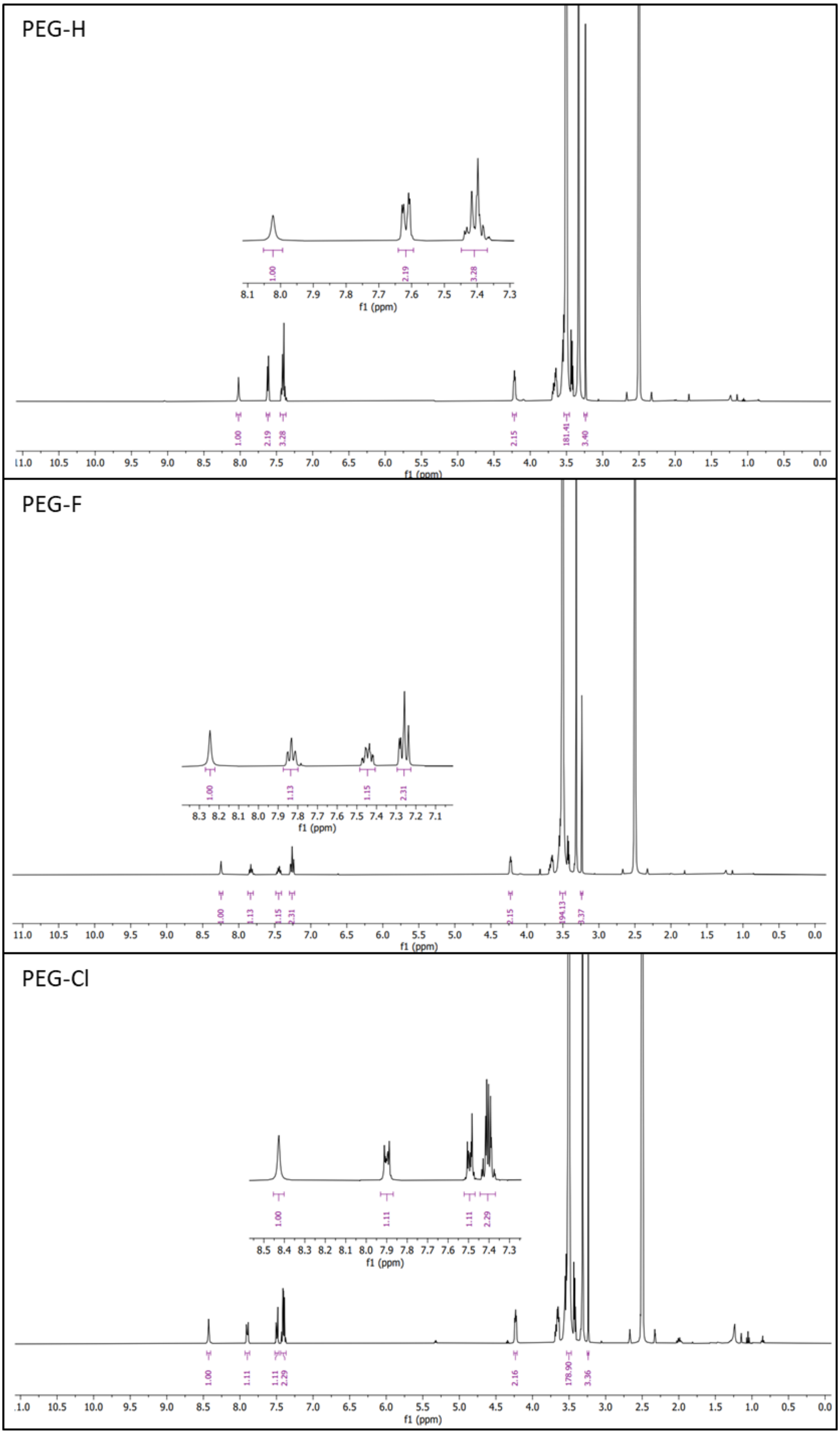
PEG-H
1.00
2.19
3.28
f1 (ppm)
2.15
181.41
3.40
f1 (ppm)
PEG-F
1.00
1.13
1.15
2.31
f1 (ppm)
2.15
194.13
3.37
f1 (ppm)
PEG-Cl
1.00
1.11
1.11
2.29
f1 (ppm)
2.16
178.90
3.36
f1 (ppm)

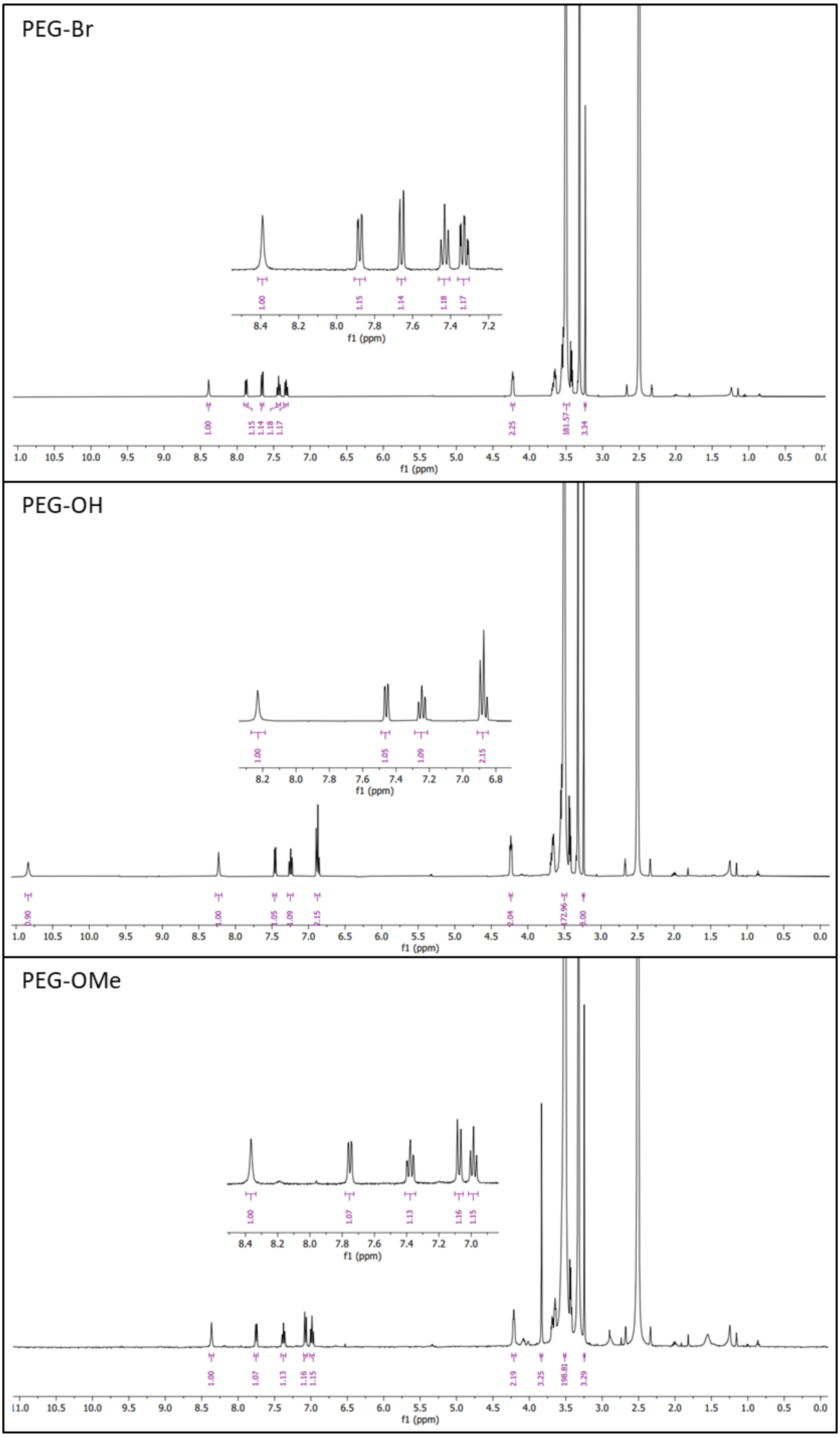

Figure S2: ^{1}H-NMR spectra of mPEG2000 acyl hydrazone model compounds featuring varying neighboring groups.

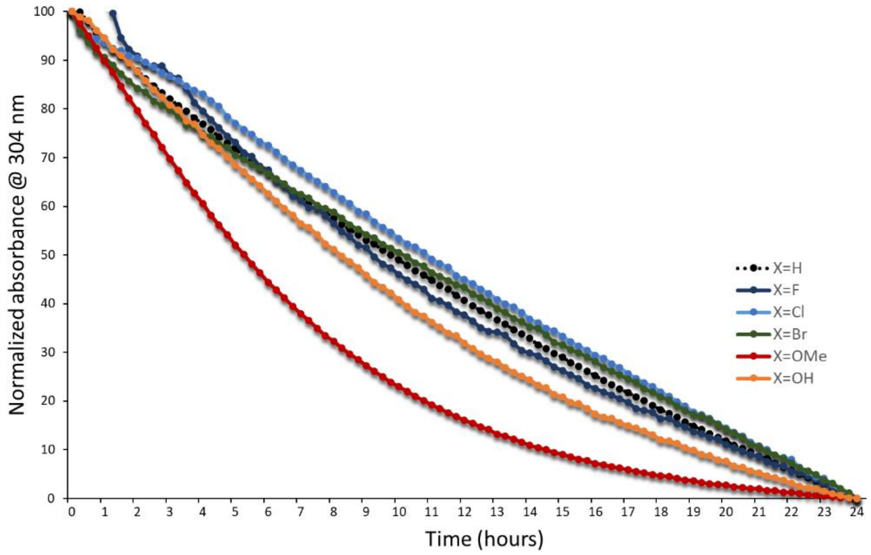

Figure S3: Kinetic of the hydrolysis of model PEG acyl hydrazone conjugates at pH 5 (normalized UV-Vis absorption monitored at 304 nm, the samples were prepared at 50 µM of acyl hydrazone conjugate).

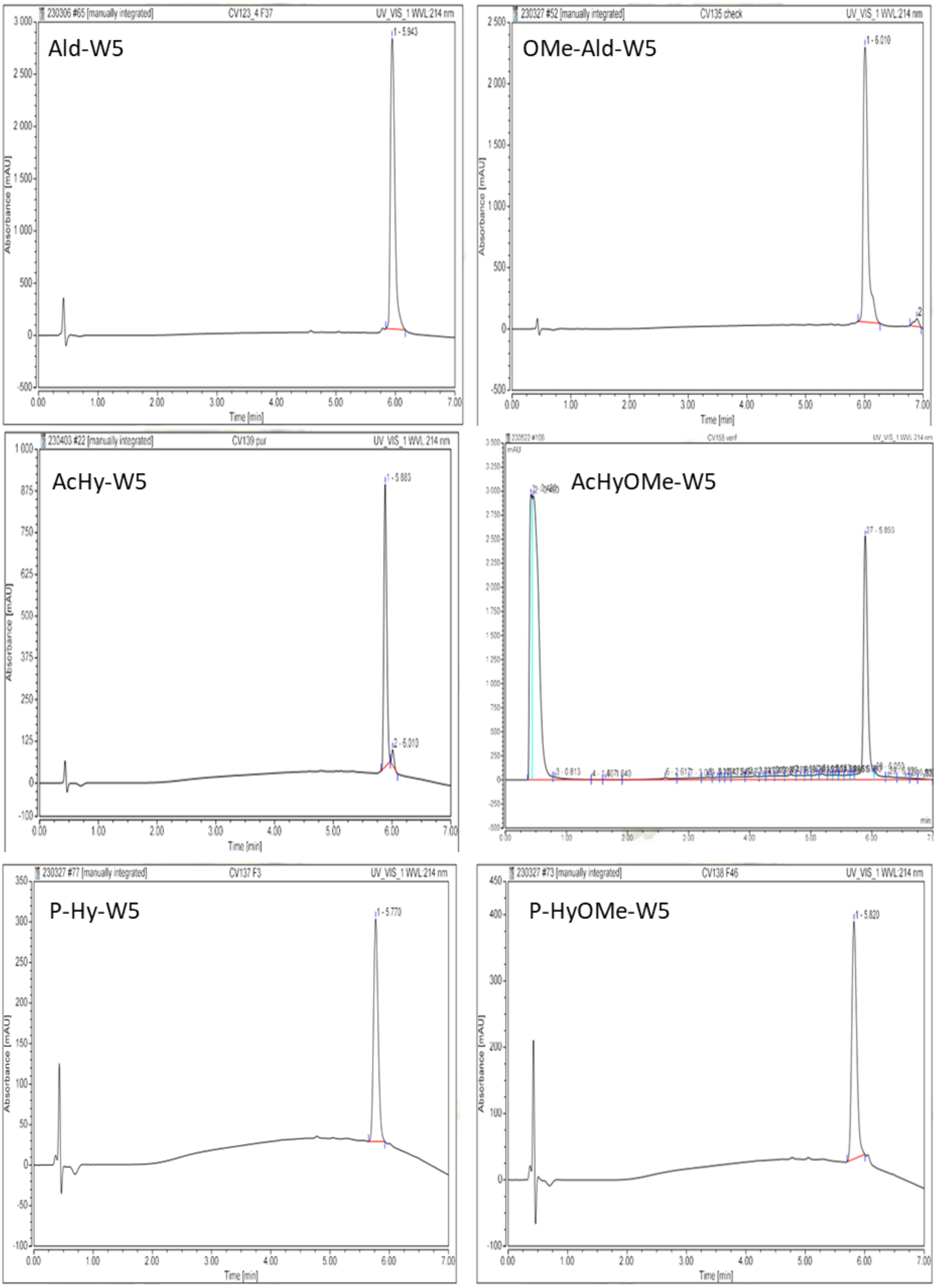

Figure S4: HPLC chromatograms of the P-Hy-W5 and P-HyOMe-W5 synthesis steps.

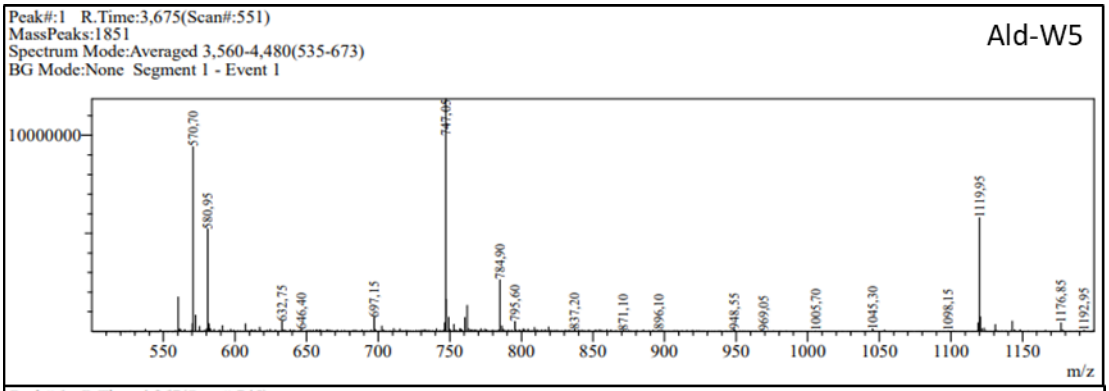
Peak#:1 R.Time:3,675(Scan#:551)
MassPeaks:1851
Spectrum Mode:Averaged 3,560-4,480(535-673)
BG Mode:None Segment 1 - Event 1
Ald-W5
10000000
570,70
580,95
632,75
646,40
697,15
747,05
784,90
795,60
837,20
871,10
896,10
948,55
969,05
1005,70
1045,30
1098,15
1119,95
1176,85
1192,95
m/z

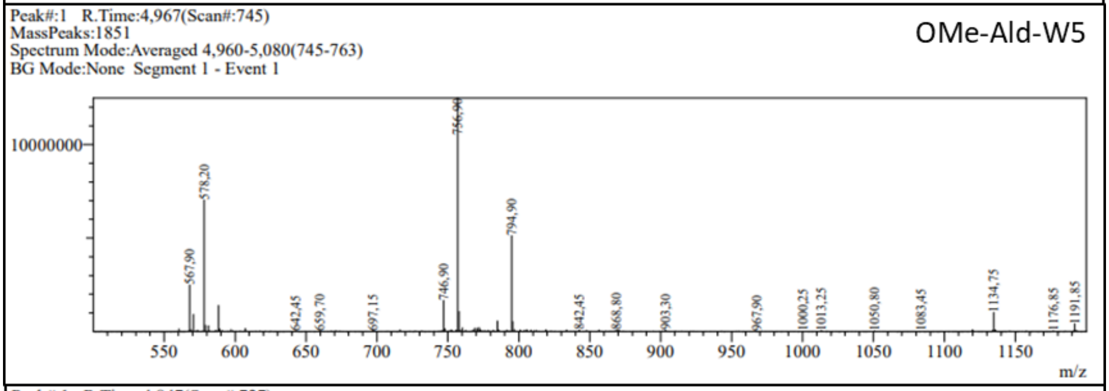
Peak#:1 R.Time:4,967(Scan#:745)
MassPeaks:1851
Spectrum Mode:Averaged 4,960-5,080(745-763)
BG Mode:None Segment 1 - Event 1
OMe-Ald-W5
10000000
567,90
578,20
642,45
659,70
697,15
746,90
756,90
794,90
842,45
868,80
903,30
967,90
1000,25
1013,25
1050,80
1083,45
1134,75
1176,85
1191,85
m/z

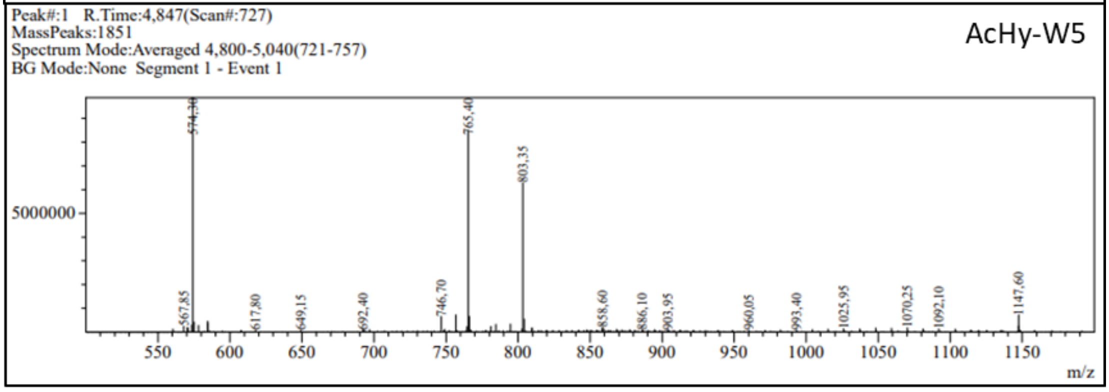
Peak#:1 R.Time:4,847(Scan#:727)
MassPeaks:1851
Spectrum Mode:Averaged 4,800-5,040(721-757)
BG Mode:None Segment 1 - Event 1
AcHy-W5
5000000
567,85
574,30
617,80
649,15
692,40
746,70
765,40
803,35
858,60
886,10
903,95
960,05
993,40
1025,95
1070,25
1092,10
1147,60
m/z

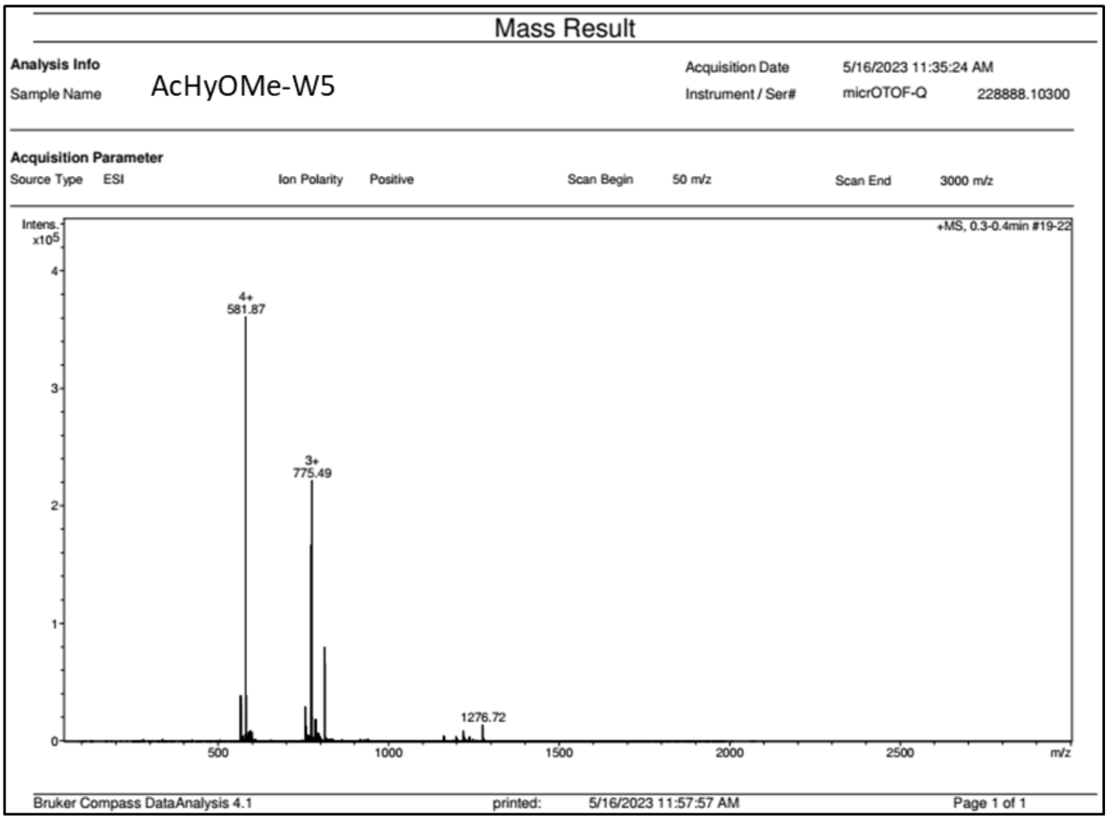
Mass Result
Analysis Info
Sample Name AcHyOMe-W5
Acquisition Date 5/16/2023 11:35:24 AM
Instrument / Ser# micrOTOF-Q 228888.10300
Acquisition Parameter
Source Type ESI
Ion Polarity Positive
Scan Begin 50 m/z
Scan End 3000 m/z
Intens. x10^5
+MS, 0.3-0.4min #19-22
4+ 581.87
3+ 775.49
1276.72
m/z
Bruker Compass DataAnalysis 4.1
printed: 5/16/2023 11:57:57 AM
Page 1 of 1

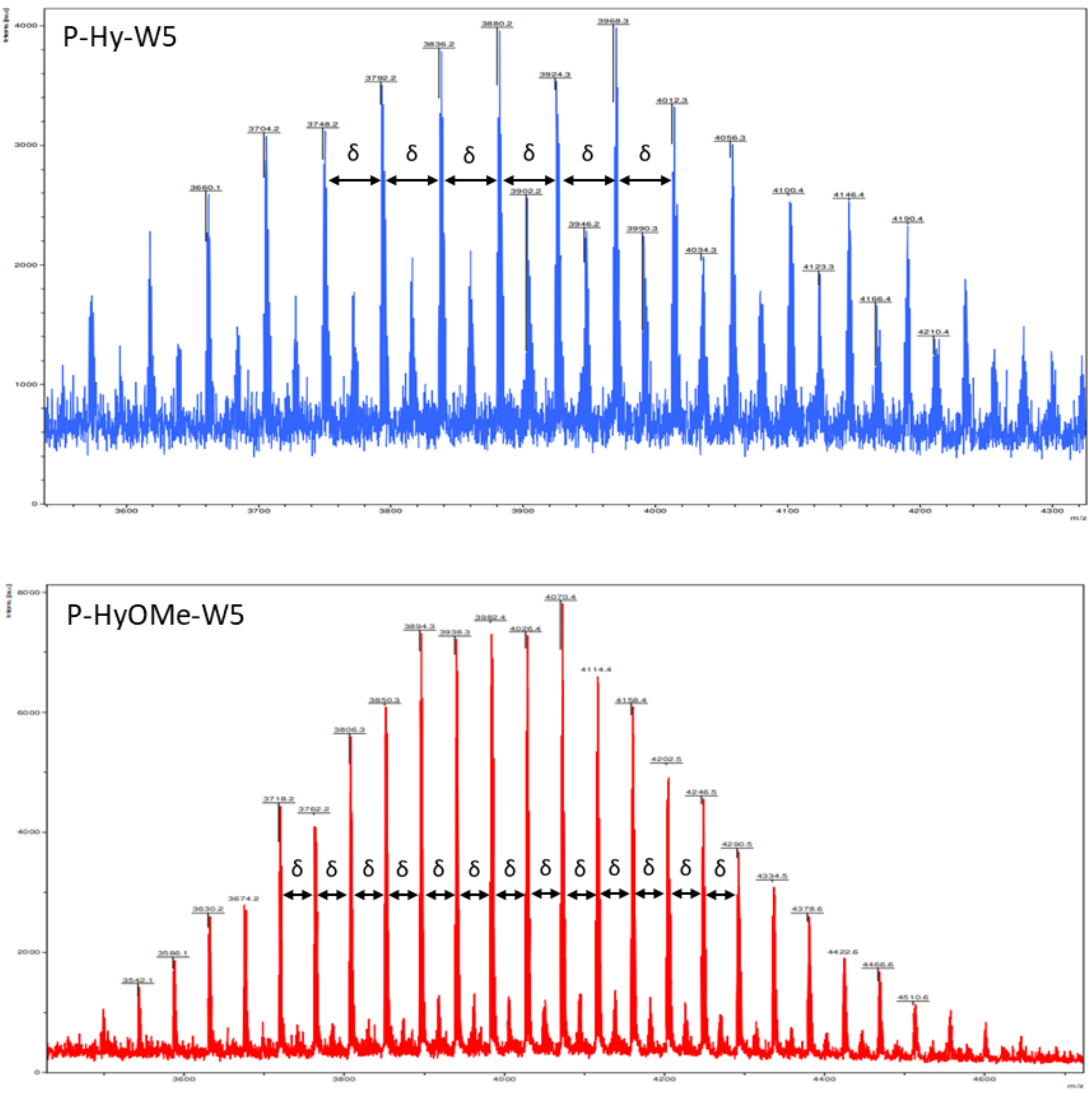

Figure S5: Mass characterization of the P-Hy-W5 and P-HyOMe-W5 synthesis steps. Extracted MS spectra of Ald-W5 and AcHy-W5; ESI-MS spectrum of AcHyOMe-W5 and MALDI spectra of P-HyW5 and P-HyOMe-W5.

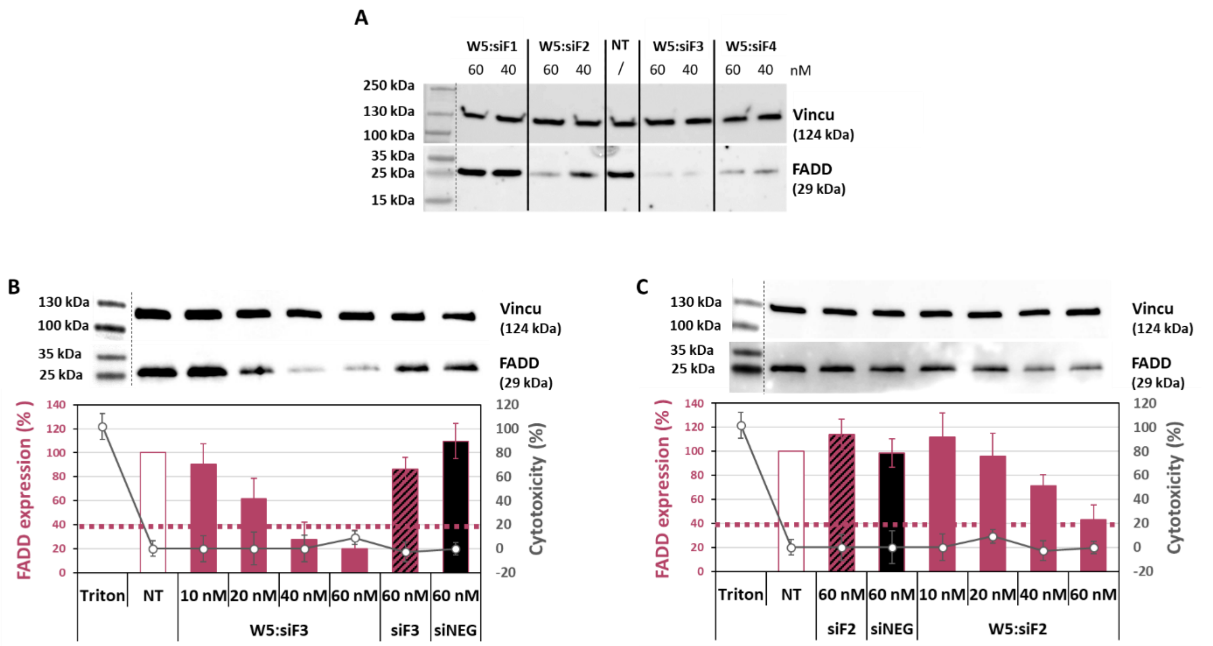

Figure S6: Evaluation of FADD silencing in human vascular endothelial cells.

(**A**) WRAP5:siRNA nanoparticles at a molar ratio of 20 (R 20) encapsulating 40 nM or 60 nM of different siRNAs targeting human FADD protein were transfected to EA.hy296 cells, and after 24 h, FADD expression was revealed by Western Blot

(**B**) WRAP5:siRNA nanoparticles at a molar ratio of 20 (R 20) encapsulating siRNA-FADD-3 (siF3) were transfected in a dose-dependent manner to EA.hy296 cells, and after 24 h, FADD expression was revealed by Western Blot

(**C**) WRAP5:siRNA nanoparticles at a molar ratio of 20 (R 20) encapsulating siRNA-FADD-2 (siF2) were transfected in a dose-dependent manner to EA.hy296 cells, and after 24 h, FADD expression was revealed by Western Blot

Signal intensities of the FADD bands were normalized to the corresponding Vinculin bands. The dashed line indicated 40% of FADD expression and the tolerated threshold of 20% cytotoxicity. Data represent the (mean ± SD) of n=2 independent experiments (each in duplicates).

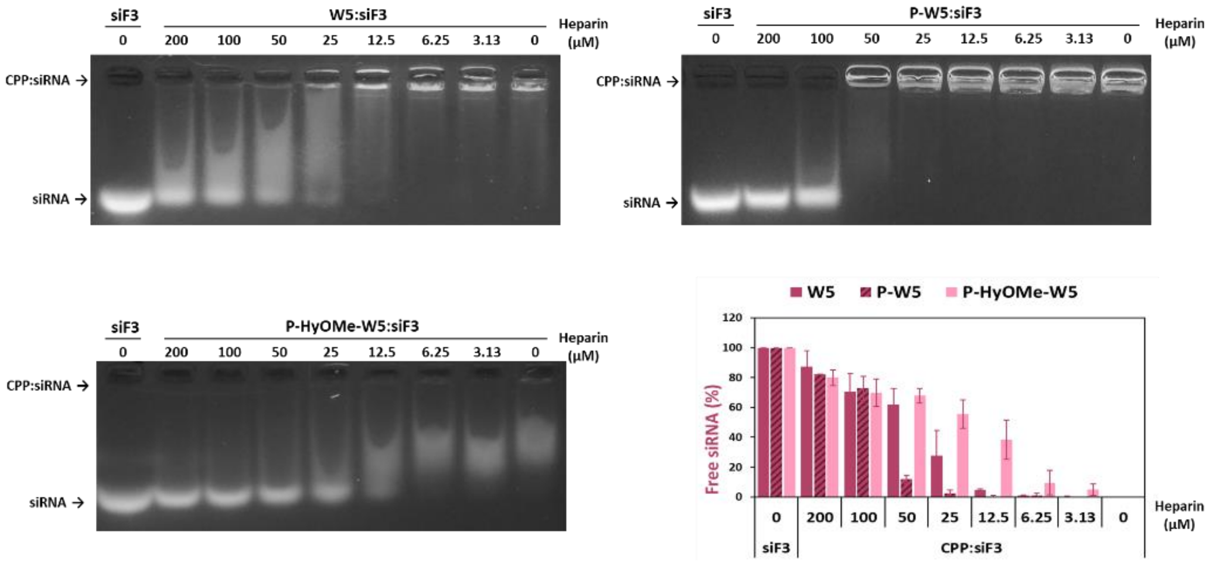

Figure S7: Evaluation of the CPP:siRNA nanoparticle stability in the presence of heparin.

The three nanoparticles W5:siF3, P-W5:siF3, and P-HyOMe-W5:siF3 were formulated (R=20, siRNA = 2.5 µM) and then incubated with increased concentrations of heparin (0 µM to 200 µM). siRNAs encapsulated in stable nanoparticles are sticking in the gel loading pocket whereas the siRNA could migrate in the gel when the nanoparticles are destabilized. The percentage of free siRNA is normalized over the siF3 condition without CPP (=100%).

A

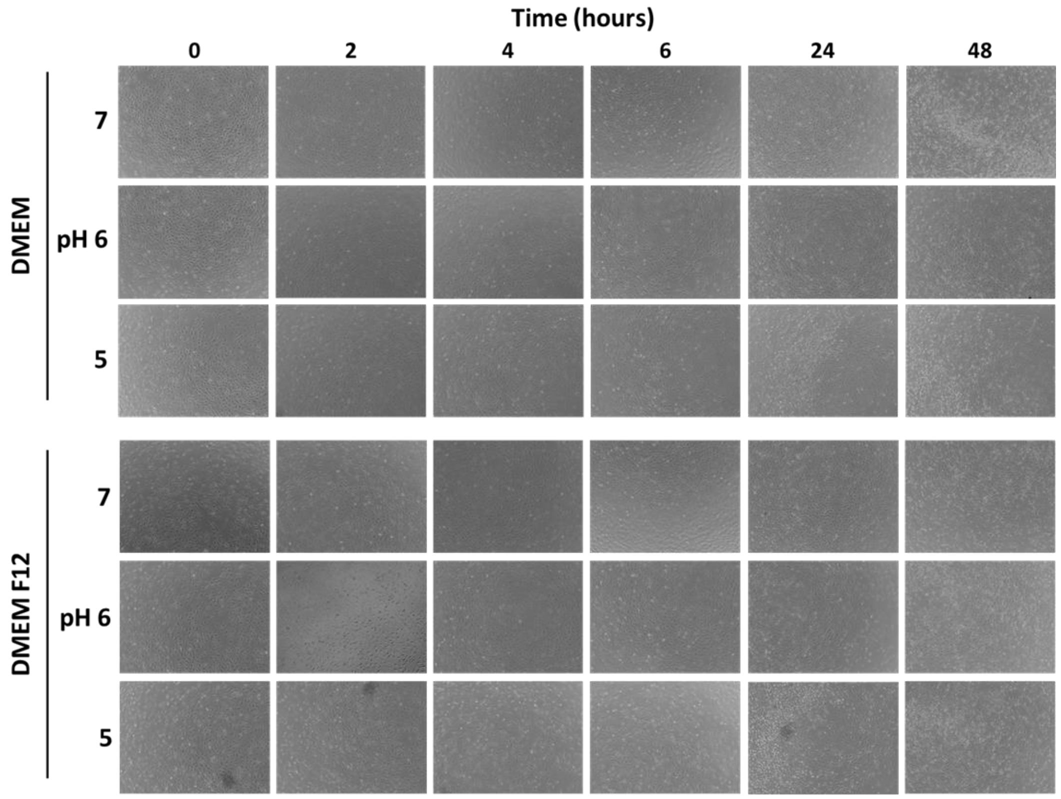

B

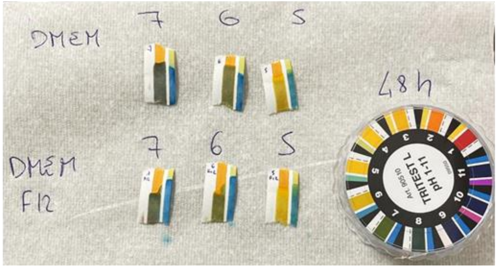

Figure S8: Evaluation of pH drop in human vascular endothelial cells.

(**A**) EA.hy926 cells were seeded in 24-well plates and after 24 h medium was exchanged at the indicated pH values. Images were acquired immediately after medium change (0 h) and then after 2 h, 4 h, 6 h, 24 h and 48 h using an Evos microscope (10x objective).

(**B**) Evaluation of the pH stability during the incubation of 48 h using a pH indicator (TRITEST L pH 1-11).

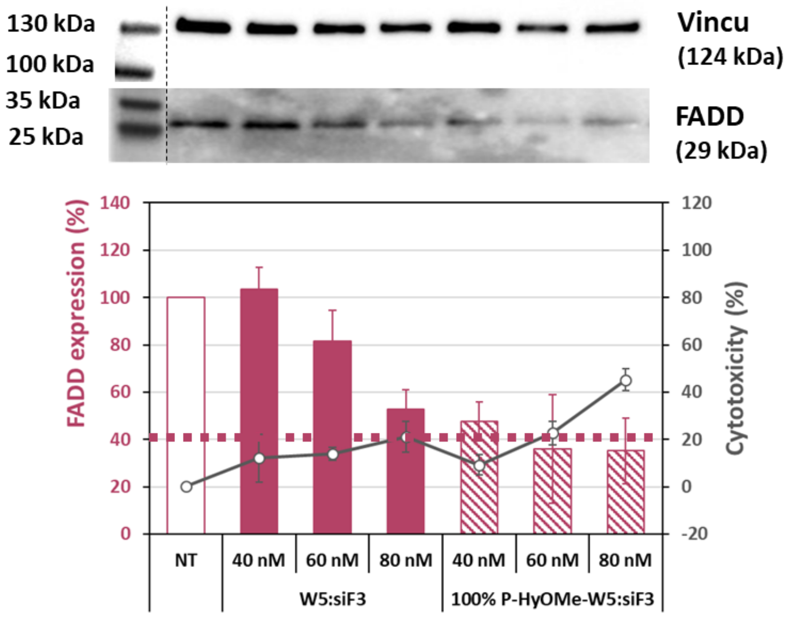

Figure S9: Evaluation of dose-depending FADD silencing in human vascular endothelial cells at pH 5.
WRAP5:siF3 or 100% P-HyOMe-W5:siF3 nanoparticles at the indicated siF3 concentrations were transfected to EA.hy296 cells at pH 5, and after 24 h, FADD expression was revealed by Western Blot.
Data represent mean ± SD, with n = 2-3 independent experiments in duplicates. Non-treated cells (NT). Transfection condition: Nanoparticles with R=20 at the indicated siRNA concentrations in the presence of 10% serum. The dashed line indicated 40% of FADD expression and the tolerated threshold of 20% cytotoxicity.

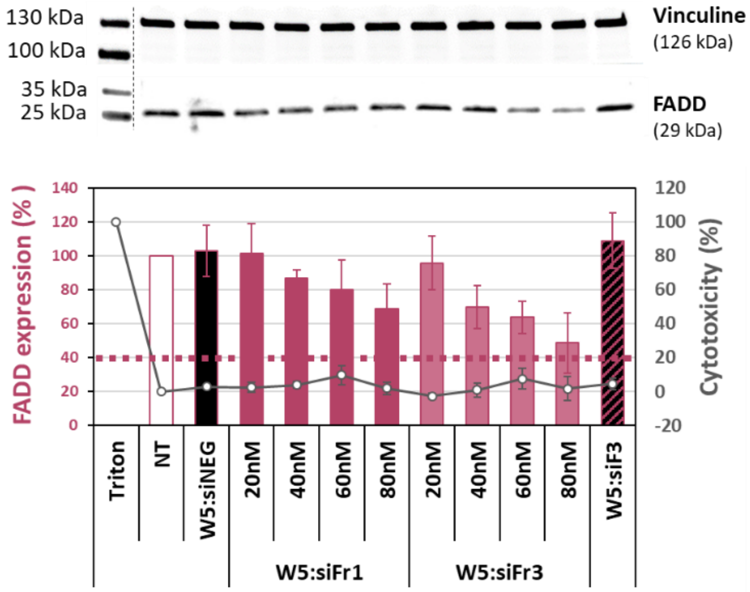

Figure S10: Evaluation of dose-depending FADD silencing in rat H9c2 cardiomyocytes.
WRAP5:siFr1 or WRAP5:siFr3 nanoparticles were transfected to rat H9c2 cardiomyocytes, and after 24 h, FADD expression was revealed by Western Blot.
Data represent mean ± SD, with n = 2 independent experiments in duplicates. Non-treated cells (NT). Transfection condition: Nanoparticles with R=20 at the indicated siRNA concentrations. The dashed line indicated 40% of FADD expression and the tolerated threshold of 20% cytotoxicity.
